# Sex and ethnic/racial-specific risk factors for gallbladder disease

**DOI:** 10.1186/s12876-017-0678-6

**Published:** 2017-12-08

**Authors:** Jane C. Figueiredo, Christopher Haiman, Jacqueline Porcel, James Buxbaum, Daniel Stram, Neal Tambe, Wendy Cozen, Lynne Wilkens, Loic Le Marchand, Veronica Wendy Setiawan

**Affiliations:** 1 Department of Preventive Medicine, Keck School of Medicine of University of Southern California, Los Angeles, California USA; 2 Samuel Oschin Comprehensive Cancer Institute, Cedars-Sinai Medical Center, Los Angeles, California USA; 3 Norris Comprehensive Cancer Center, Keck School of Medicine of University of Southern California, Los Angeles, California USA; 4 Department of Medicine, Keck School of Medicine, University of Southern California, Los Angeles, California USA; 5 Department of Pathology, Keck School of Medicine, University of Southern California, Los Angeles, California USA; 6 Epidemiology Program, University of Hawaii Cancer Center, Honolulu, Hawaii USA

**Keywords:** Gallbladder, Stones, Cholecystectomy, Ethnicity/race

## Abstract

**Background:**

Gallbladder disease (GBD) is a highly prevalent condition; however, little is known about potential differences in risk factors by sex and ethnicity/race. Our aim was to evaluate dietary, reproductive and obesity-related factors and GBD in multiethnic populations.

**Methods:**

We performed a prospective analysis from the Multiethnic Cohort study who self-identified as non-Hispanic White (*n* = 32,103), African American (*n* = 30,209), Japanese (*n* = 35,987), Native Hawaiian (*n* = 6942) and Latino (*n* = 39,168). GBD cases were identified using Medicare and California hospital discharge files (1993–2012) and self-completed questionnaires. We used exposure information on the baseline questionnaire to identify exposures of interest. Associations were estimated by hazard ratios and 95% confidence intervals using Cox models adjusted for confounders.

**Result:**

After a median 10.7 years of follow-up, there were 13,437 GBD cases. BMI over 25 kg/m^2^, diabetes, past and current smoking, red meat consumption, saturated fat and cholesterol were significant risk factors across ethnic/racial populations (p-trends < 0.01). Protective factors included vigorous physical activity, alcohol use, fruits, vegetables and foods rich in dietary fiber (p-trends < 0.01). Carbohydrates were inversely associated with GBD risk only among women and Latinos born in South America/Mexico (p-trend < 0.003). Parity was a significant risk factor among women; post-menopausal hormones use was only associated with an increased risk among White women (estrogen-only: HR = 1.24; 95% CI = 1.07–1.43 and estrogen + progesterone: HR = 1.23; 95% CI = 1.06–1.42).

**Conclusion:**

Overall, dietary, reproductive and obesity-related factors are strong risk factors for GBD affecting men and women of different ethnicities/races; however some risk factors appear stronger in women and certain ethnic groups.

**Electronic supplementary material:**

The online version of this article (doi: 10.1186/s12876-017-0678-6) contains supplementary material, which is available to authorized users.

## Background

Gallbladder disease (GBD) is a highly prevalent condition affecting up to 15% of the adult U.S. population and is a leading cause of hospital admissions [1]. In developed countries, GBD occurs largely as a result of formation of cholesterol gallstones. While most gallstones are clinically silent, 20% of people harboring stones experience biliary symptoms that at some point require surgical removal of the gallbladder [2]. Over 700,000 cholecystectomies are performed annually in the U.S. at a cost of approximately $6.5 billion [3].

The basis for GBD is multifactorial including infections, genetic susceptibility and modifiable lifestyle factors. Of particular note is the significant difference in rates of GBD by ethnicity/race and geography. The prevalence is highest among Hispanic populations of Central and South American and in individuals with Native American ancestry [4]. In the U.S., the prevalence of GBD is also notably higher in Hispanics compared to any other ethnic/racial group [5]. Genetic factors in part explain some of the observed racial differences in incidence; large population-based twin studies have estimated that genetic effects account for 25% (95% CI = 9–40%) [6]. Other factors may explain a larger fraction of the attributable risk associated with GBD, including lifestyle factors related to obesity and diet.

Diet constitutes a major source of cholesterol and several factors directly related to diet including high caloric intake and low dietary fiber intake, have been identified as risk factors for gallstone formation in both population-based studies and experimental animal models [7]. Although there exist inconsistencies across studies, diets high in vegetables, fruits and total fiber have been shown to reduce risk of GBD, and diets high in animal protein, carbohydrates and cholesterol increase risk [8–10]. Obesity, particularly abdominal or centripetal obesity, has also been observed as a risk factor for gallstone disease in some studies in the U.S. [11], Europe [12] and China [13]; other studies have observed no association in Mexicans [14] and Japanese [15] populations. Lifestyle behaviors that help maintain body weight including increased physical activity appear to lower risk in some [13, 16–18], but not all studies [19, 20]. Other behaviors including smoking and alcohol use have also been inconsistently associated with GBD risk [21–26], and may depend on patient characteristics including sex, ethnicity/race and country.

Several studies have documented a disproportionate number of women diagnosed with GBD compared to men. Overall, women are almost twice as likely as men to form gallstones, undergo cholecystectomy and to be diagnosed with gallbladder cancer [3], but these sex differences tends to narrow with increasing age [27]. The sex disparity has been attributed to hormonal factors, in particular estrogen. Several observational studies in non-Hispanic Whites have observed a modest increased risk of GBD associated with use of oral contraceptives [28] and post-menopausal hormones [29, 30]. Overall, parity appears to be the most consistent reproductive risk factor and has been observed in studies in the U.S. [31, 32].

In this study, we examined the association between dietary, reproductive and obesity-related factors and GBD by sex and ethnicity/race: African Americans, Japanese Americans, Native Hawaiians, Latinos and Whites, in the Multiethnic Cohort (MEC) study.

## Methods

### Study population

The MEC is an ongoing population-based prospective cohort study with over 215,000 men and women from Hawaii and California assembled between 1993 and 1996. The details of the study design and baseline characteristics have been published [33]. Briefly, the cohort is comprised predominantly of African Americans, Native Hawaiians, Japanese Americans, Latinos, and Whites (aged 45–75 years). The baseline mailed questionnaire assessed diet, lifestyle, anthropometrics, family and personal medical history, and for women menstrual, reproductive history and exogenous hormone use. The MEC participants older than 65 years were linked to Centers for Medicare Services (CMS) claims (1999–2012) using Social Security number, sex, and date of birth, and 93% of these participants were successfully linked [34]. California participants were also linked to the Office of Statewide Health Planning and Development Hospital Discharge Data (1993–2012).

For this study, we excluded participants (*N* = 22,824) who were not from the five major ethnic groups or who had missing baseline information on the established GBD risk factors and important covariates (i.e. smoking status, body mass index, diabetes, alcohol intake, and education level). Participants with a diagnosis of gallbladder cancer identified via tumor registries (*N* = 8) or GBD identified via baseline questionnaire or the California hospital discharge data (*N* = 1426) before cohort entry were excluded. Hawaii participants who were not Medicare members (N = 14,035) or who were not fee-for-service (FFS) members were excluded (*N* = 28,438), as we had no opportunity to discover a GBD diagnosis in this group. A total of 144,409 eligible participants were available for analysis.

### GBD case identification

We considered individuals with gallstones [International Classification of Diseases, 9^th^ Revision (ICD-9), code 574.x], cholelithiasis (575.x) or those who had undergone a cholecystectomy (procedure codes 51.2× and CPT codes 47,480, 47,490, 47,562 47,563, 47,564, 47,600, 47,605, 47,610, 47,612, 47,620, 56,340, 56,341, 56,342) as cases with GBD. Cases were identified from one or more claims in the MedPAR (hospitalization) or the CHDD or two or more claims if they were from outpatient files. We identified 13,513 GBD cases through December 31, 2012; 76 cases were excluded because we could not identify eligible non-cases for the risk set.

### Exposure assessment

Data on demographic factors and known/potential risk factors for GBD including anthropometry, alcohol intake, smoking history, physician-diagnosed type 2 diabetes, physical activity, and dietary factors were obtained from the MEC baseline questionnaire (available in Additional file 1). Body mass index (BMI) was calculated as weight in kg divided by height in m^2^ and categorized as <25, 25- < 30, and ≥30 kg/m^2^. Vigorous activity (hours/day) were categorized using quartile distributions in the cohort. Alcohol intake was categorized as non-drinkers, < 24, 24- ≤ 48, and > 48 g ethanol/day. Smoking status was categorized as never, past, and current, and for past and current smoking, they were further stratified by < 20 and ≥ 20 pack years. Dietary factors and nutrients (e.g. red meat, fruits, vegetables, fiber, saturated fat, cholesterol, and carbohydrate, etc.) were categorized using overall quartile distributions in the cohort. The reference period for the dietary factors was the past year.

### Statistical analysis

We used the date of the first GBD claim as the event date. As the time of diagnosis was not measured precisely, but was based on claim date, we used a Cox proportional hazards model for interval data based on a logistic model with a complementary log-log link. For each case, we constructed the set of at-risk individuals (alive and without a GBD diagnosis at the date of index case’s diagnosis) matched on ethnicity, sex, exact birth year, study area, and if a case was identified via Medicare, length of Medicare coverage (±1 year). The associations between risk factors and GBD were estimated by hazard ratio (HR) and its 95% confidence interval (CI) adjusted for education, BMI, history of diabetes, smoking status and pack-years, and alcohol intake. Further adjustment for caloric intake was done for the diet analysis. Tests for trend were performed by entering the ordinal values representing categories of exposures as continuous variables in the models. Statistical analyses were performed with SAS 9.3 software (SAS Institute, Inc., Cary, NC). All *p*-values were two sided.

## Results

After a mean 10.7 years of follow-up (SD = 5.0), there were 13,437 incident cases of GBD among the 144,409 at-risk cohort participants (Table 1). Overall the mean age at diagnosis was 73.5 (range = 45.3–94.8) years. The majority of cases of GBD were reports of gallstone only (40.2%) and 49.2% of all cases had a cholecystectomy. GBD cases included more women (57.9%) than men (42.1%). The cases’ ethnicity breakdown was 35.2% Latinos, 23.3% Japanese, 19.4% whites, 18.4% African Americans, and 3.8% Native Hawaiians. The characteristics and distributions of selected risk factors by ethnicity/race are shown in Additional file 2: Table S1.

**Table 1:** Characteristics of study participants in the Multiethnic Cohort

	Total *N* = 144,409		GBD cases *N* = 13,437	
	N	%	N	%
Age at GBD occurrence, years				
Mean (range)			73.5 (45.3–94.8)	
Age at cohort entry, years				
< 50	19,307	13.4	758	5.6
50–54	22,658	15.7	1483	11.0
55–59	25,808	17.9	2542	18.9
60–64	26,559	18.4	2992	22.3
65–69	25,739	17.8	3053	22.7
≥ 70	24,338	16.9	2609	19.4
Gender				
Men	64,901	44.9	5658	42.1
Women	79,508	55.1	7779	57.9
Race/ethnicity				
White	32,103	22.2	2600	19.4
African American	30,209	20.9	2469	18.4
Native Hawaiian	6942	4.8	511	3.8
Japanese American	35,987	24.9	3133	23.3
Latino – US born	20,405	14.1	2398	17.9
Latino – Mexico/South America born	18,763	13.0	2326	17.3
Area				
Hawaii	51,579	35.7	4100	30.5
California	92,830	64.3	9337	69.5
Type				
Gallstones only			5395	40.2
Cholecystitis only			927	6.9
Cholecystectomy only			123	0.9
Gallstones and Cholecystitis			507	3.8
Gallstones and Cholecystectomy			3882	28.9
Cholecystitis and Cholecystectomy			505	3.8
Gallstones, Cholecystitis, and Cholecystectomy			2098	15.6
Year of diagnosis^a,^ ^b^				
1993–1997			1352	10.1
1998–2002			3362	25.0
2003–2007			4243	31.6
2008–2012			4480	33.3

^a^Gallbladder disease included gallstone (574.XX), cholecystitis (575.XX), and cholecystectomy (procedure codes 51.2X and CPT codes 47,480, 47,490, 47,562 47,563, 47,564, 47,600, 47,605, 47,610, 47,612, 47,620, 56,340, 56,341, 56,342), whichever came first

^b^Cases diagnosed before 1999 were identified from CHDD; between 1999 and 2012 were from both CHDD and Medicare

Both overweight and obese men and women were at significantly higher risk compared to those with a BMI under 25 kg/m^2^ (p-trends < 0.0001, Table 2) with no differences by ethnicity/race (Table 3). Concomitant and past diabetes was associated with GBD risk in men (HR = 1.44; 95% CI = 1.34–1.55) and women (HR = 1.46; 95% CI = 1.37–1.56). Diabetes was also consistently associated with a higher risk of GBD in all ethnic/racial groups (*p*-values ≤ 0.0257). Past and current smoking were also significantly associated with risk of GBD among men and women (HR range = 1.09–1.37; with increasing RRs with increasing pack years; p-trends < 0.0001). By ethnicity/race, current smoking (> 20 pack-years) was associated with a significant increased risk in Whites, African Americans and Japanese Americans and US-born Latinos (p-trends ≤ 0.0055). There was a non-significant increased risk between past or current smoking and GBD among Native Hawaiians; and only past smoking > 20 pack-years were significantly associated with GBD in Mexican/SA-born Latinos (HR = 1.38; 1.12–1.69). Daily consumption of alcohol significantly reduced the risk of GBD among men and women (p-trends ≤ 0.0001). Vigorous physical activity was also inversely associated with GBD risk in all categories for men and women compared to no activity (p-trends ≤ 0.004). The highest level of vigorous activity was associated with a reduced the risk of GBD among all groups; the estimates for the trend were non-significant in Native Hawaiians, Japanese and US-born Latinos, but in the same direction.

**Table 2:** Associations between modifiable risk factors and GBD risk by sex

	Men		Women	
Risk Factors	No. Cases	HR^a^(95% CI)	No. Cases	HR^a^(95% CI)
BMI (kg/m^2^)				
< 25	1577	1.00 (ref.)	2463	1.00 (ref.)
25 - < 30	2612	1.23 (1.15–1.31)	2444	1.33 (1.25–1.41)
≥ 30	1076	1.55 (1.43–1.69)	2213	1.74 (1.63–1.85)
*P trend*		< .0001		< .0001
Diabetes				
No	4360	1.00 (ref.)	5968	1.00 (ref.)
Yes	905	1.44 (1.34–1.55)	1152	1.46 (1.37–1.56)
Smoking (pack-years)				
Never	1537	1.00 (ref.)	4003	1.00 (ref.)
Past, < 20	2013	1.09 (1.01–1.16)	1740	1.10 (1.04–1.16)
Past, ≥ 20	888	1.16 (1.07–1.27)	418	1.30 (1.17–1.44)
Current, < 20	411	1.16 (1.04–1.30)	582	1.17 (1.08–1.28)
Current, ≥ 20	416	1.22 (1.09–1.37)	377	1.37 (1.23–1.53)
*P trend*		< .0001		< .0001
Alcohol intake (ethanol g/day)				
0	2185	1.00 (ref.)	4755	1.00 (ref.)
< 24	2256	0.92 (0.86–0.97)	2083	0.86 (0.82–0.91)
24- ≤ 48	501	0.85 (0.77–0.94)	190	0.80 (0.69–0.93)
> 48	323	0.86 (0.76–0.97)	92	0.92 (0.74–1.14)
*P trend*		0.0001		< .0001
Vigorous Activity (hrs/day)				
0	2063	1.00 (ref.)	4567	1.00 (ref.)
> 0- ≤ 0.21	892	0.89 (0.82–0.96)	1078	0.90 (0.84–0.97)
> 0.21- ≤ 0.46	831	0.86 (0.79–0.94)	740	0.95 (0.87–1.03)
> 0.46	1479	0.80 (0.74–0.86)	735	0.90 (0.83–0.98)
*P trend*		< .0001		0.0041
Parity^b^				
0 children			771	1.00 (ref.)
1 child			746	1.09 (0.98–1.20)
2–3 children			2906	1.05 (0.97–1.14)
≥ 4 children			2899	1.14 (1.05–1.23)
*P trend*				0.0036
Age at First Live Birth (years)^b^				
No children			771	1.00 (ref.)
≤ 20			2505	1.10 (1.01–1.20)
21–30			3613	1.07 (0.99–1.15)
> 30			433	1.05 (0.94–1.19)
*P trend*				0.5200
Ever Used Birth Control^b^				
No			4714	1.00 (ref.)
Yes			2608	1.05 (0.99–1.11)
Menopausal Hormone Use^b^				
Never users			3383	1.00 (ref.)
Past users			1442	0.97 (0.91–1.04)
Current estrogen-only			1113	1.09 (1.02–1.17)
Current estrogen + progesterone			1099	1.06 (0.99–1.14)

^a^HR stratified by set number (defined by matching factors: birth year, sex, ethnicity, study area, duration of Medicare enrollment—for cases identified from Medicare) and adjusted for smoking-pack years (never, past < 20, past ≥ 20, current < 20, current ≥ 20), alcohol intake (0, < 24, 24- ≤ 48, > 48 ethanol g/day), body mass index (< 25, 25- < 30, 30+ kg/m^2^), diabetes (no/yes), and education (≤ High School, some college, ≥college graduate). Menopausal hormone use limited to postmenopausal women

^b^Females only

**Table 3:** Association between modifiable risk factors and GBD (HR^a^ and 95% CI) by race/ethnicity

	NH-White *N* = 2600	African American *N* = 2469	Native Hawaiian *N* = 511	Japanese American *N* = 3133	Latino US born *N* = 2398	Latino Mexico/SA born *N* = 2326
BMI (kg/m^2^)						
< 25	1.00 (ref.)	1.00 (ref.)	1.00 (ref.)	1.00 (ref.)	1.00 (ref.)	1.00 (ref.)
25 - < 30	1.30 (1.19–1.43)	1.21 (1.08–1.36)	1.27 (0.99–1.63)	1.37 (1.26–1.48)	1.20 (1.08–1.34)	1.29 (1.15–1.44)
≥ 30	1.68 (1.50–1.87)	1.56 (1.39–1.75)	1.62 (1.26–2.10)	1.73 (1.51–1.99)	1.56 (1.39–1.75)	1.74 (1.54–1.97)
*P trend*	<.0001	<.0001	0.0002	<.0001	<.0001	<.0001
Diabetes						
No	1.00 (ref.)	1.00 (ref.)	1.00 (ref.)	1.00 (ref.)	1.00 (ref.)	1.00 (ref.)
Yes	1.35 (1.16–1.57)	1.58 (1.43–1.76)	1.33 (1.04–1.72)	1.27 (1.14–1.42)	1.57 (1.41–1.74)	1.47 (1.31–1.64)
Smoking (pack-years)						
Never	1.00 (ref.)	1.00 (ref.)	1.00 (ref.)	1.00 (ref.)	1.00 (ref.)	1.00 (ref.)
Past, < 20	1.03 (0.93–1.14)	1.17 (1.06–1.29)	1.00 (0.80–1.25)	1.11 (1.02–1.22)	1.12 (1.01–1.23)	1.07 (0.96–1.19)
Past, ≥ 20	1.10 (0.97–1.24)	1.32 (1.13–1.55)	0.93 (0.68–1.28)	1.34 (1.19–1.51)	1.09 (0.91–1.31)	1.38 (1.11–1.71)
Current, <20	1.33 (1.11–1.59)	1.26 (1.10–1.44)	1.06 (0.75–1.51)	1.05 (0.87–1.27)	1.10 (0.94–1.29)	1.11 (0.95–1.30)
Current, ≥ 20	1.30 (1.12–1.50)	1.50 (1.27–1.77)	0.87 (0.61–1.24)	1.3 (1.1–1.54)	1.38 (1.12–1.69)	0.90 (0.66–1.21)
*P trend*	<.0001	<.0001	0.5831	<.0001	0.0055	0.1849
Alcohol intake (ethanol g/day)						
0	1.00 (ref.)	1.00 (ref.)	1.00 (ref.)	1.00 (ref.)	1.00 (ref.)	1.00 (ref.)
< 24	0.86 (0.78–0.94)	0.89 (0.81–0.98)	0.94 (0.76–1.16)	0.93 (0.86–1.01)	0.89 (0.81–0.98)	0.84 (0.76–0.93)
24- ≤ 48	0.82 (0.71–0.95)	0.94 (0.76–1.16)	1.05 (0.71–1.56)	0.83 (0.7–0.99)	0.77 (0.63–0.94)	0.74 (0.57–0.94)
> 48	0.83 (0.69–1.00)	1.16 (0.93–1.46)	0.77 (0.45–1.32)	0.70 (0.52–0.93)	0.82 (0.65–1.03)	0.87 (0.65–1.16)
*P trend*	0.0011	0.5926	0.4816	0.0016	0.0016	0.0008
Vigorous activity (hrs/day)						
0	1.00 (ref.)	1.00 (ref.)	1.00 (ref.)	1.00 (ref.)	1.00 (ref.)	1.00 (ref.)
> 0- ≤ 0.21	0.91 (0.81–1.03)	0.96 (0.85–1.07)	1.20 (0.91–1.59)	0.91 (0.82–1.01)	0.87 (0.77–0.99)	0.79 (0.69–0.91)
> 0.21- ≤ 0.46	0.88 (0.77–1.00)	0.80 (0.69–0.93)	1.10 (0.83–1.46)	1.05 (0.94–1.18)	0.94 (0.82–1.07)	0.79 (0.68–0.92)
> 0.46	0.79 (0.71–0.89)	0.68 (0.59–0.80)	0.96 (0.75–1.24)	0.92 (0.82–1.03)	0.97 (0.86–1.09)	0.80 (0.71–0.91)
*P trend*	<.0001	<.0001	0.7369	0.3474	0.4939	0.0001
Parity^b^						
0 children	1.00 (ref.)	1.00 (ref.)	1.00 (ref.)	1.00 (ref.)	1.00 (ref.)	1.00 (ref.)
1 child	1.12 (0.91–1.37)	1.13 (0.94–1.35)	1.26 (0.61–2.60)	1.04 (0.84–1.29)	1.19 (0.88–1.62)	0.89 (0.66–1.20)
2–3 children	1.11 (0.95–1.31)	1.01 (0.86–1.19)	0.97 (0.55–1.72)	1.14 (0.97–1.34)	1.00 (0.79–1.27)	0.89 (0.70–1.12)
≥ 4 children	1.09 (0.91–1.29)	1.12 (0.95–1.32)	1.14 (0.65–1.99)	1.14 (0.94–1.37)	1.15 (0.92–1.45)	1.08 (0.87–1.33)
*P trend*	0.4436	0.3186	0.6047	0.0987	0.1931	0.0851
Age at first live birth (years)^b^						
No children	1.00 (ref.)	1.00 (ref.)	1.00 (ref.)	1.00 (ref.)	1.00 (ref.)	1.00 (ref.)
≤ 20	1.20 (1.00–1.44)	1.04 (0.89–1.22)	1.19 (0.68–2.10)	1.09 (0.86–1.38)	1.10 (0.87–1.39)	0.99 (0.79–1.23)
21–30	1.07 (0.91–1.25)	1.06 (0.90–1.24)	0.94 (0.54–1.65)	1.15 (0.98–1.34)	1.07 (0.85–1.34)	0.99 (0.80–1.23)
> 30	0.98 (0.76–1.27)	1.14 (0.87–1.50)	1.03 (0.40–2.67)	1.11 (0.89–1.38)	1.10 (0.76–1.60)	0.97 (0.72–1.30)
*P trend*	0.6757	0.3744	0.2289	0.1600	0.8651	0.9194
Ever used birth control^b^						
No	1.00 (ref.)	1.00 (ref.)	1.00 (ref.)	1.00 (ref.)	1.00 (ref.)	1.00 (ref.)
Yes	1.11 (0.98–1.25)	1.01 (0.89–1.13)	0.80 (0.59–1.08)	1.10 (0.96–1.25)	1.02 (0.89–1.17)	1.06 (0.92–1.21)
Menopausal Hormone Use^b^						
Never users	1.00 (ref.)	1.00 (ref.)	1.00 (ref.)	1.00 (ref.)	1.00 (ref.)	1.00 (ref.)
Past users	0.94 (0.81–1.10)	0.95 (0.84–1.08)	0.90 (0.63–1.28)	1.03 (0.88–1.20)	1.09 (0.93–1.27)	0.89 (0.75–1.04)
Current estrogen only	1.24 (1.07–1.43)	1.14 (0.98–1.34)	0.83 (0.55–1.24)	1.06 (0.92–1.23)	0.94 (0.79–1.13)	1.14 (0.92–1.40)
Current estrogen + progesterone	1.23 (1.06–1.42)	1.14 (0.93–1.39)	0.85 (0.57–1.27)	0.97 (0.84–1.11)	1.07 (0.88–1.28)	0.96 (0.76–1.21)

^a^HR stratified by set number (defined by matching factors: birth year, ethnicity, study area, duration of Medicare enrollment—for cases identified from Medicare) and adjusted for smoking-pack years (never, past <20, past ≥20, current <20, current ≥20), alcohol intake (0, <24, 24- ≤ 48, >48 ethanol g/day), body mass index (<25, 25- < 30, 30+ kg/m2), diabetes (no/yes), and education (≤ High School, some college, ≥college graduate)

^b^Females only

Among women, selected reproductive factors were evaluated as risk factors for GBD (Table 2). The risk associated with giving birth to 4 or more children was 14% higher compared to nulliparity (HR = 1.14; 95% CI = 1.05–1.23, p-trend = 0.0036). Women who reported having their first child prior to age 20 were at higher risk of GBD (HR = 1.10; 95% CI = 1.01–1.20) compared to those who never had children. Ever use of oral contraceptives was not associated with GBD; age at first use and duration of use did not modify this association (data not shown). Current use of estrogen-only menopausal hormones was also associated with GBD risk (HR = 1.09; 95% CI: 1.02–1.17). Post-menopausal estrogen and progesterone use was only marginally significant (HR = 1.06; 95% CI = 0.99–1.14). By ethnicity/race, these associations were limited to White women (HR = 1.24; 95% CI = 1.07–1.43 and HR = 1.23; 95% CI = 1.06–1.42, respectively, Table 3).

Significant trend in reducing risk was observed for increasing intake of vegetables, fruits and fiber-rich foods among men and women (p-trends < 0.0001, Table 4). Among women, increasing quartiles of carbohydrates were inversely associated with GBD risk (HR quartile 4 vs. quartile 1 = 0.86; 95% CI = 0.80–0.93, *p*-trend < 0.0001); no association was observed in men. Conversely, significant increased risk of GBD was observed for increasing amount of red meat, and foods rich in saturated fat and cholesterol (p-trends < 0.0001). We observed consistently significant estimates of risk with GBD among Whites, African-Americans and Latinos for dietary fiber, vegetables, fruits, red meat, saturated fat and cholesterol (Table 5). Carbohydrates were not associated with GBD risk, except for Latinos from Mexico/South America (HR quartile 4 vs. quartile 1 = 0.82; 9%CI = 0.71–0.93, p-trend = 0.0028). Among Native Hawaiians and Japanese Americans we observed no statistically significant findings, except for a decreased risk of GBD with dietary fiber (p-trend = 0.015) and increased risk associated with red meat intake (p-trend = 0.0024) and saturated fat (p-trend = 0.045) among Japanese Americans.

**Table 4:** Associations between selected dietary factors and GBD by sex

	Men		Women	
	No. Cases	HR^a^(95% CI)	No. Cases	HR^a^(95% CI)
Dietary Fiber (g/1000 kcal/day)				
≤ 9.0	1723	1.00 (ref.)	1409	1.00 (ref.)
> 9.0 – ≤ 11.6	1587	1.00 (0.93–1.08)	1912	0.96 (0.90–1.04)
> 11.6 – ≤ 14.8	1300	0.91 (0.84–0.98)	2142	0.88 (0.81–0.94)
> 14.8	1048	0.80 (0.74–0.88)	2316	0.81 (0.75–0.87)
*P trend*		< .0001		< .0001
Total Vegetables (g/1000 kcal/day)				
≤ 109.3	1744	1.00 (ref.)	1569	1.00 (ref.)
> 109.3 – ≤ 150.1	1582	1.00 (0.93–1.07)	1788	0.96 (0.89–1.03)
> 150.1 – ≤ 202.6	1298	0.89 (0.82–0.96)	2084	0.96 (0.89–1.02)
> 202.6	1034	0.85 (0.78–0.92)	2338	0.89 (0.83–0.95)
*P trend*		< .0001		0.0007
Total Fruits (g/1000 kcal/day)				
≤ 78.9	1741	1.00 (ref.)	1591	1.00 (ref.)
> 78.9 – ≤ 145.9	1558	0.93 (0.87–1.00)	1723	0.91 (0.84–0.97)
> 145.9 – ≤ 239.8	1307	0.89 (0.82–0.96)	2128	0.93 (0.87–1.00)
> 239.8	1052	0.93 (0.85–1.01)	2337	0.85 (0.80–0.91)
*P trend*		0.0216		<.0001
Red Meat (g/1000 kcal/day)				
≤ 14.3	1022	1.00 (ref.)	2087	1.00 (ref.)
> 14.3 – ≤ 24.4	1305	1.07 (0.98–1.17)	2057	1.08 (1.01–1.15)
> 24.4 – ≤ 36.1	1528	1.08 (0.99–1.17)	1860	1.13 (1.06–1.21)
> 36.1	1803	1.13 (1.04–1.23)	1775	1.23 (1.15–1.31)
*P trend*		0.0055		<.0001
% Calories from Saturated Fat				
≤ 7.1	1269	1.00 (ref.)	1878	1.00 (ref.)
> 7.1 – ≤ 8.9	1433	1.12 (1.04–1.21)	1875	1.02 (0.96–1.10)
> 8.9 – ≤ 10.8	1423	1.09 (1.00–1.18)	1962	1.11 (1.04–1.19)
> 10.8	1533	1.14 (1.04–1.24)	2064	1.20 (1.12–1.29)
*P trend*		0.0134		< .0001
Cholesterol (mg/1000 kcal/day)				
≤ 77.8	1241	1.00 (ref.)	1925	1.00 (ref.)
> 77.8 – ≤ 101.7	1383	1.06 (0.98–1.15)	1937	1.06 (0.99–1.13)
> 101.7 – ≤ 128.7	1456	1.08 (1.00–1.17)	1966	1.14 (1.06–1.22)
> 128.7	1578	1.06 (0.98–1.15)	1951	1.22 (1.14–1.31)
*P trend*		0.1823		< .0001
% Calories from Carbohydrate				
≤ 46.1	1619	1.00 (ref.)	1676	1.00 (ref.)
> 46.1 – ≤ 52.1	1494	1.00 (0.93–1.08)	1907	0.94 (0.88–1.01)
> 52.1 – ≤ 58.2	1327	0.98 (0.90–1.06)	2083	0.91 (0.85–0.97)
> 58.2	1218	0.99 (0.91–1.09)	2113	0.86 (0.80–0.93)
*P trend*		0.7258		< .0001

^a^HR stratified by set number (defined by matching factors: birth year, sex, ethnicity, study area, duration of Medicare enrollment) and adjusted for smoking-pack years (never, past <20, past ≥20, current <20, current ≥20), alcohol intake (0, <24, 24- ≤ 48, >48 ethanol g/day), body mass index (<25, 25- < 30, 30+ kg/m2), diabetes (no/yes), calories, and education (≤ High School, some college, ≥college graduate)

**Table 5:** Associations between selected dietary factors and GBD (HR^a^ and 95% CI) by race/ethnicity

	NH-White N = 2600	African American N = 2469	Native Hawaiian N = 511	Japanese American N = 3133	Latino US born N = 2398	Latino Mexico/SA born N = 2326
Dietary Fiber (g/1000 kcal/day)						
≤ 9.0	1.00 (ref.)	1.00 (ref.)	1.00 (ref.)	1.00 (ref.)	1.00 (ref.)	1.00 (ref.)
> 9.0 – ≤ 11.6	0.91 (0.82–1.03)	0.90 (0.80–1.01)	0.84 (0.66–1.07)	1.04 (0.94–1.14)	1.11 (0.97–1.27)	0.98 (0.83–1.16)
> 11.6 – ≤ 14.8	0.92 (0.82–1.03)	0.82 (0.73–0.93)	0.79 (0.60–1.03)	0.99 (0.89–1.10)	0.94 (0.82–1.08)	0.80 (0.68–0.94)
> 14.8	0.75 (0.66–0.85)	0.78 (0.68–0.88)	0.83 (0.62–1.12)	0.85 (0.76–0.96)	0.89 (0.77–1.02)	0.78 (0.67–0.91)
*P trend*	< .0001	< .0001	0.1166	0.0146	0.0033	< .0001
Total Vegetables (g/1000 kcal/day)						
≤ 109.3	1.00 (ref.)	1.00 (ref.)	1.00 (ref.)	1.00 (ref.)	1.00 (ref.)	1.00 (ref.)
> 109.3 – ≤ 150.1	1.03 (0.92–1.15)	0.99 (0.89–1.10)	0.91 (0.70–1.18)	1.02 (0.92–1.13)	0.89 (0.79–1.00)	0.94 (0.82–1.09)
> 150.1 – ≤ 202.6	0.91 (0.81–1.02)	0.88 (0.78–0.99)	1.02 (0.78–1.33)	0.98 (0.88–1.09)	0.91 (0.81–1.03)	0.92 (0.80–1.05)
> 202.6	0.86 (0.76–0.97)	0.88 (0.78–0.99)	0.94 (0.71–1.23)	0.93 (0.83–1.04)	0.83 (0.74–0.94)	0.85 (0.74–0.97)
*P trend*	0.0024	0.0094	0.8386	0.1317	0.0079	0.0085
Total Fruits (g/1000 kcal/day)						
≤ 78.9	1.00 (ref.)	1.00 (ref.)	1.00 (ref.)	1.00 (ref.)	1.00 (ref.)	1.00 (ref.)
> 78.9 – ≤ 145.9	0.85 (0.76–0.95)	0.83 (0.74–0.94)	1.03 (0.80–1.33)	0.93 (0.84–1.04)	1.08 (0.96–1.21)	0.89 (0.78–1.00)
> 145.9 – ≤ 239.8	0.81 (0.72–0.91)	0.92 (0.82–1.04)	0.96 (0.73–1.26)	0.94 (0.85–1.05)	1.02 (0.90–1.15)	0.90 (0.79–1.02)
> 239.8	0.85 (0.76–0.96)	0.83 (0.74–0.94)	0.76 (0.57–1.02)	0.93 (0.84–1.04)	1.02 (0.90–1.16)	0.78 (0.69–0.88)
*P trend*	0.0076	0.0245	0.0586	0.3021	0.9962	0.0002
Red Meat (g/1000 kcal/day)						
≤ 14.3	1.00 (ref.)	1.00 (ref.)	1.00 (ref.)	1.00 (ref.)	1.00 (ref.)	1.00 (ref.)
> 14.3 – ≤ 24.4	1.06 (0.95–1.18)	1.12 (1.00–1.27)	0.88 (0.66–1.18)	1.06 (0.96–1.18)	1.08 (0.94–1.23)	1.10 (0.97–1.25)
> 24.4 – ≤ 36.1	1.18 (1.06–1.32)	1.08 (0.95–1.22)	0.91 (0.69–1.22)	1.15 (1.04–1.27)	1.06 (0.93–1.21)	1.09 (0.96–1.24)
> 36.1	1.13 (1.00–1.28)	1.32 (1.17–1.49)	1.20 (0.91–1.58)	1.16 (1.04–1.30)	1.15 (1.01–1.30)	1.13 (1.00–1.28)
*P trend*	0.0100	< .0001	0.1033	0.0024	0.0545	0.0934
% Calories from Saturated Fat						
≤ 7.1	1.00 (ref.)	1.00 (ref.)	1.00 (ref.)	1.00 (ref.)	1.00 (ref.)	1.00 (ref.)
> 7.1 – ≤ 8.9	1.02 (0.91–1.15)	1.03 (0.90–1.18)	1.16 (0.91–1.49)	1.07 (0.98–1.17)	1.11 (0.95–1.29)	1.08 (0.94–1.25)
> 8.9 – ≤ 10.8	1.07 (0.95–1.20)	1.08 (0.95–1.23)	1.01 (0.77–1.32)	1.11 (1.00–1.23)	1.18 (1.02–1.36)	1.14 (0.99–1.30)
> 10.8	1.13 (1.00–1.27)	1.18 (1.04–1.35)	1.06 (0.78–1.42)	1.09 (0.93–1.27)	1.20 (1.04–1.39)	1.27 (1.11–1.46)
*P trend*	0.0361	0.0039	0.9779	0.0452	0.0104	0.0003
Cholesterol (mg/1000 kcal/day)						
≤ 77.8	1.00 (ref.)	1.00 (ref.)	1.00 (ref.)	1.00 (ref.)	1.00 (ref.)	1.00 (ref.)
> 77.8 – ≤ 101.7	1.05 (0.94–1.18)	1.09 (0.95–1.26)	0.94 (0.72–1.23)	1.09 (1.00–1.19)	1.09 (0.96–1.25)	1.04 (0.91–1.18)
> 101.7 – ≤ 128.7	1.08 (0.97–1.21)	1.19 (1.04–1.36)	1.15 (0.88–1.50)	1.03 (0.93–1.14)	1.24 (1.10–1.41)	1.08 (0.96–1.23)
> 128.7	1.18 (1.05–1.33)	1.20 (1.05–1.36)	1.30 (0.99–1.70)	1.10 (0.98–1.23)	1.16 (1.02–1.32)	1.14 (1.01–1.30)
*P trend*	0.0069	0.0043	0.0252	0.1768	0.0115	0.0271
% Calories from Carbohydrate						
≤ 46.1	1.00 (ref.)	1.00 (ref.)	1.00 (ref.)	1.00 (ref.)	1.00 (ref.)	1.00 (ref.)
> 46.1 – ≤ 52.1	0.99 (0.88–1.10)	0.89 (0.80–0.99)	0.89 (0.66–1.20)	1.03 (0.89–1.19)	1.12 (1.00–1.25)	0.88 (0.78–1.00)
> 52.1 – ≤ 58.2	0.94 (0.84–1.06)	0.92 (0.82–1.03)	0.92 (0.69–1.24)	1.00 (0.86–1.15)	1.01 (0.90–1.15)	0.84 (0.74–0.96)
> 58.2	0.90 (0.80–1.03)	0.89 (0.78–1.01)	0.93 (0.69–1.26)	0.96 (0.83–1.11)	1.01 (0.88–1.16)	0.82 (0.71–0.93)
*P trend*	0.0891	0.0767	0.8459	0.2587	0.8483	0.0028

^a^HR stratified by matching factors (birth year, ethnicity, study area, duration of Medicare enrollment) and adjusted for smoking-pack years (never, past < 20, past ≥ 20, current < 20, current ≥ 20), alcohol intake (0, < 24, 24- ≤ 48, > 48 ethanol g/day), body mass index (< 25, 25- < 30, 30+ kg/m2), diabetes (no/yes), calories, and education (≤ High School, some college, ≥ college graduate

In a sensitivity analysis excluding cases who had a cholecystectomy without a report of gallstone or cholecystitis, we observed similar results (data not shown).

## Discussion

These findings from the large population-based MEC, suggest that several lifestyle factors increase the risk of GBD in both men and women across different ethnic/racial groups. Our observations are consistent with previous observational studies with much smaller sample sizes, provide better estimation of risk associated with various factors, and importantly compare risks across understudied minorities in the U.S. Results from the Third National Health and Nutrition Examination Survey study was limited to three ethnic/racial populations and selected lifestyle factors over a shorter duration of follow-up and did not report on dietary factors [35]. Here we obtained stronger evidence for the role of dietary factors in GBD across all ethnic/racial groups, except Native Hawaiians. In addition, we confirm known associations between obesity, diabetes, physical activity, smoking and parity across ethnic/racial groups. We did not observe appreciable differences in the associations by sex or ethnicity/race, except for an increased risk of post-menopausal hormone use in White women.

Cholesterol hypersaturation of the bile and cholesterol nucleation leading to dysmotility have been implicated in the pathogenesis of GBD [36]. These observations have led to intense investigation of the roles of specific dietary components in gallbladder physiology and gallstone formation. Although results have not been entirely consistent across studies likely due to methodological differences, the overall body of evidence supports the hypothesis that a diet characterized by high caloric intake, refined carbohydrates, animal protein and cholesterol and low in vegetables and dietary fiber increase risk of GBD [8–10]. Early ecological studies noted an increased prevalence of GBD associated with diets characterized by higher intake of fat and refined carbohydrate and lower dietary fiber in the U.S. and Japan [37, 38]. In the Nurses’ Health Study of primarily non-Hispanic women, fruit and vegetable consumption were significantly inversely associated with risk of cholecystectomy [39]. Data in other ethnic/racial group are limited; a study from the Third National Health and Nutrition Examination Survey among Mexican Americans investigated dietary patterns and risk of GBD finding distinct differences by sex but no significant associations with GBD risk [40].

Obesity and associated co-morbidities, strongly linked with poor diet and positive energy balance, have also been recognized as important risk factors for GBD. In the Nurses’ Health Study among largely non-Hispanic White women, obese participants (BMI ≥ 30 kg/m^2^) had a 2-fold excess risk and extremely obese women (BMI ≥ 45 kg/m^2^) had a 7-fold increased risk of symptomatic gallstones compared to women with a BMI < 24 kg/m^2^ [41]. While studies in China [13], Mexico [14] and Japan [15] have reported variable results, although the overall summary estimates have suggested an increased risk associated with obesity. We also observed a strong association between type II diabetes and GBD risk across ethnic/racial populations. In agreement, the Strong Heart Study also reported that diabetes was associated with GBD in women; however their analysis did not confirm the association among men [35]. In agreement with our results, low physical activity, high body-mass-index and diabetes were found to increase GBD risk in most [13, 16–18, 42], but not all studies [19, 20].

Smoking is a known risk factor for diseases in several organs, including those not directly exposed to inhaled smoke, including the gastrointestinal tract. In the gallbladder, smoking is suspected to lead to disruption in emptying, which is thought to be associated with gallstone formation. Both bile stasis and disrupted gallbladder motility are important factors for gallstone formation. Several studies have investigated the association between smoking and GBD with inconsistent results; among Japanese men and women some studies [21, 22], but not all [23, 24] have observed an increased risk associated with active smoking; similar inconsistencies have been observed among Europeans [25, 26]. In terms of alcohol use, most previous studies in the U.S., Europe and Japan have found an inverse association between alcohol intake and GBD [15, 21, 22, 43–45]. Moderate alcohol intake may protect against gallstone development through its association with reduced biliary cholesterol saturation and higher serum HDL [46]. Overall our results across a large sample size of diverse populations provide confirmatory evidence of the increased risk associated with smoking and decreased risk associated with alcohol use.

Sex disparities in the incidence of several diseases have been reported. For GBD, the rates reported are 1.5–3-times higher in women compared to men [47]. Possible explanations for these discrepancies may be due to sex hormones and potentially other differences in dietary patterns and tobacco and alcohol exposures or other lifestyle-related behaviors. Overall, in agreement with our study, pregnancy has been found to be the most consistent risk factor for GBD in previous studies [48], in particular among overweight or obese women [49]. Pregnancy is a critical time period of increased risk of insulin resistance (gestational diabetes) [50] as well as biliary sludge, a suspected to be a potential precursor to gallstones. Biliary sludge, a mixture of cholesterol and calcium bilirubinate crystals in bile, develops in up to 30% of women [31] and likely represents a precursor of gallstones [51]. We observed the highest risk of GBD among women who reported having a child before age 20. No other reproductive variables were significant in our study, except for post-menopausal use among White women only. A previous meta-analysis found highly inconsistent results across studies for oral contraceptives; the summary estimate was RR = 1.36 (95% CI = 1.15–1.62) [28]. Secondary analysis of randomized clinical trials of post-menopausal hormones among largely non-Hispanic Whites have observed significantly elevated risk of GBD among women randomized to the treatment arm compared to placebo; in the Heart and Estrogen/progestin Replacement Study, RR (estrogen + progestin) =1.38 (95% CI, 1.00–1.92) [29] and the Women’s Health Initiative, RR (estrogen-only) = 1.67 (95% CI = 1.35–2.06) and RR (estrogen + progestin) = 1.59 (95% CI = 1.28–1.97) [30].

Our study has several strengths and some limitations. To date no other study has been able to compare risk estimates across sex and ethnicity/race in a single study with uniform data collection on risk factors collected up to 19 years prior to diagnosis. In addition, the MEC has been shown to be representative of the populations represented in the cohort [33], and thus our results are broadly generalizable to U.S. populations. We also excluded subjects with GBD at baseline to investigate prospectively risk factors for incident GBD. One limitation is that our analysis is based on exposure data collected from self-reported questionnaires; however measurement error is likely to be non-differential. Furthermore, we defined GBD as any occurrence of gallstones, cholelithiasis and cholecystectomy from claim records. The validity of the algorithm to accurately define cases has not been evaluated in the MEC; however, in our own sensitivity analysis comparing results obtained with our broad definition of GBD with those obtained using claims of cholecystectomy alone, we did not observe significant differences in the results.

## Conclusion

Our study represents the first prospective analysis from multiethnic US populations with varying exposure levels across men and women by ethnicity/race and risk for GBD. Our results strongly support a substantial role of several lifestyle factors in the development of GBD across diverse populations.

## Additional files

Additional file 1: The Los Angeles Cancer Research Survey. MEC questionnaire for collecting baseline data used in this study. (PDF 5195 kb)

Additional file 2: Table S1. Distribution of Risk Factors among MEC participants by sex and race/ethnicity. This table shows the distribution of risk factors in the MEC by sex and race/ethnicity. (DOCX 16 kb)

## Abbreviations

BMI: Body Mass Index
CI: Confidence interval
GBD: Gallbladder disease
HR: Hazard ratio
MEC: Multiethnic cohort study
