# Supplementary material for: Sex and ethnic/racial-specific risk factors for gallbladder disease
Source: BMC Gastroenterol. 2017 Dec 8;17:153. doi: 10.1186/s12876-017-0678-6 (PMC5723039; doi:10.1186/s12876-017-0678-6)
Supplement: Supplementary file 1 — The Los Angeles Cancer Research Survey. MEC questionnaire for collecting baseline data used in this study. (PDF 5195 kb) [file 12876_2017_678_MOESM1_ESM.pdf]

the Los Angeles Cancer Research Survey

Norris Cancer Center
University of Southern California
School of Medicine

Is the address label correct? ________

If not, please give the correct spelling of your name and your correct address.

NAME ________________________________

STREET ________________________________

CITY ________________ STATE ________ ZIP ________

This survey is to be filled out **ONLY** by the person named on the address label.

If you have questions, please call (213) 224-5007.

MARKING INSTRUCTIONS

- **Use Pencil only.**
  One is provided for your use.

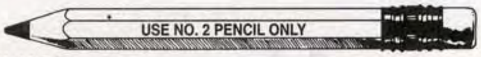

- **Do NOT use ink or ballpoint pens.**
- **Fill in the circle completely, staying within the circle line.**

  CORRECT MARK ● ○ ○ ○ INCORRECT MARKS

- **Erase cleanly any answer you wish to change.**
- **Do NOT make any stray marks in this booklet.**
- **Marking Examples:**

If you are a female, you would fill in the circle like this:

SEX
○ Male
● Female

If your place of birth is Canada, which is not listed, you would fill in the circle for "other" and write your answer in the box like this:

PLACE OF BIRTH
○ USA (includes Hawai'i)
○ Mexico
○ Central or South America
○ Europe
○ Africa
○ Cuba or Caribbean Islands
○ China, Hong Kong, or Taiwan
○ Japan (includes Okinawa)
○ Korea
○ Philippines
● Other: *(write in)*

Canada

KEEP HANDWRITING WITHIN THE BOX

Sometimes questions are designed to help you line up numbers in certain columns. For questions like this, first write the numbers in the boxes, then fill in the correct circle in each column.

If your birthdate is February 2, 1906, you would write the day and the year in the boxes, and then fill in the circle for February, the circles 0 and 2 for the day, and the circles 0 and 6 for the year.

MONTH	DAY		YEAR			
	0	2	1	9	0	6
○ JAN	●	⓪			●	⓪
● FEB	①	①			①	①
○ MAR	②	●			②	②
○ APR	③	③			③	③
○ MAY		④			④	④
○ JUN		⑤			⑤	⑤
○ JUL		⑥			⑥	●
○ AUG		⑦			⑦	⑦
○ SEP		⑧			⑧	⑧
○ OCT		⑨			⑨	⑨
○ NOV						
○ DEC						

PLEASE BEGIN THIS SURVEY ON PAGE 1.

Copyright © 1993 All Rights Reserved Worldwide.

BACKGROUND INFORMATION

1. **WHAT IS YOUR SEX?**
   - ◯ Male
   - ◯ Female

2. **WHAT IS YOUR CURRENT MARITAL STATUS?**
   - ◯ Married
   - ◯ Separated
   - ◯ Divorced
   - ◯ Widowed
   - ◯ Never Married

3. **WHERE WERE YOU BORN?**
   - ◯ USA (includes Hawai'i)
   - ◯ Mexico
   - ◯ Central or South America
   - ◯ Europe
   - ◯ Africa
   - ◯ Cuba or Caribbean Islands
   - ◯ China, Hong Kong, or Taiwan
   - ◯ Japan (includes Okinawa)
   - ◯ Korea
   - ◯ Philippines
   - ◯ Other: *(write in)* ____________

4. **HOW MANY YEARS HAVE YOU LIVED IN THE UNITED STATES?**
   - ◯ 5 years or less
   - ◯ 6 - 10 years
   - ◯ 11 - 15 years
   - ◯ 16 - 25 years
   - ◯ 26 years or more

5. **WHERE WAS YOUR MOTHER BORN?**
   - ◯ USA (includes Hawai'i)
   - ◯ Mexico
   - ◯ Central or South America
   - ◯ Europe
   - ◯ Africa
   - ◯ Cuba or Caribbean Islands
   - ◯ China, Hong Kong, or Taiwan
   - ◯ Japan (includes Okinawa)
   - ◯ Korea
   - ◯ Philippines
   - ◯ Other: *(write in)* ____________

6. **WHERE WAS YOUR FATHER BORN?**
   - ◯ USA (includes Hawai'i)
   - ◯ Mexico
   - ◯ Central or South America
   - ◯ Europe
   - ◯ Africa
   - ◯ Cuba or Caribbean Islands
   - ◯ China, Hong Kong, or Taiwan
   - ◯ Japan (includes Okinawa)
   - ◯ Korea
   - ◯ Philippines
   - ◯ Other: *(write in)* ____________

7. **WHAT IS YOUR ETHNIC OR RACIAL BACKGROUND? *(Mark all that apply)***
   - ◯ Black or African-American
   - ◯ Chinese
   - ◯ Filipino
   - ◯ Hawaiian
   - ◯ Japanese (includes Okinawan)
   - ◯ Korean
   - ◯ Mexican or other Hispanic
   - ◯ White or Caucasian
   - ◯ Other: *(write in)* ____________

8. **WHAT IS YOUR MOTHER'S ETHNIC OR RACIAL BACKGROUND? *(Mark all that apply)***
   - ◯ Black or African-American
   - ◯ Chinese
   - ◯ Filipino
   - ◯ Hawaiian
   - ◯ Japanese (includes Okinawan)
   - ◯ Korean
   - ◯ Mexican or other Hispanic
   - ◯ White or Caucasian
   - ◯ Other: *(write in)* ____________

9. **WHAT IS YOUR FATHER'S ETHNIC OR RACIAL BACKGROUND? *(Mark all that apply)***
   - ◯ Black or African-American
   - ◯ Chinese
   - ◯ Filipino
   - ◯ Hawaiian
   - ◯ Japanese (includes Okinawan)
   - ◯ Korean
   - ◯ Mexican or other Hispanic
   - ◯ White or Caucasian
   - ◯ Other: *(write in)* ____________

10. **HOW MANY CHILDREN HAVE YOU HAD?**
(Include any who may have died.)

- ○ 0
- ○ 1
- ○ 2
- ○ 3
- ○ 4
- ○ 5
- ○ 6
- ○ 7 or more

11. **HOW MANY FULL SISTERS DO YOU HAVE?**
(Include any who may have died.)

- ○ 0
- ○ 1
- ○ 2
- ○ 3
- ○ 4
- ○ 5
- ○ 6
- ○ 7 or more

12. **HOW MANY FULL BROTHERS DO YOU HAVE?**
(Include any who may have died.)

- ○ 0
- ○ 1
- ○ 2
- ○ 3
- ○ 4
- ○ 5
- ○ 6
- ○ 7 or more

13. **HOW MUCH DO YOU CURRENTLY WEIGH?**
(Record weight in pounds <u>or</u> kilograms)

POUNDS			OR	KILOGRAMS		
0	0	0		0	0	0
1	1	1		1	1	1
2	2	2		2	2	2
3	3	3			3	3
4	4	4			4	4
	5	5			5	5
	6	6			6	6
	7	7			7	7
	8	8			8	8
	9	9			9	9

14. **HOW MUCH DID YOU WEIGH AT AGE 21?**
(Record weight in pounds <u>or</u> kilograms)

POUNDS			OR	KILOGRAMS		
0	0	0		0	0	0
1	1	1		1	1	1
2	2	2		2	2	2
3	3	3			3	3
4	4	4			4	4
	5	5			5	5
	6	6			6	6
	7	7			7	7
	8	8			8	8
	9	9			9	9

15. **HOW TALL ARE YOU?**
(Record height in feet/inches <u>or</u> centimeters)

FEET	INCHES	OR	CENTIMETERS		
	0		0	0	0
	1		1	1	1
	2		2	2	2
3	3			3	3
4	4			4	4
5	5			5	5
6	6			6	6
7	7			7	7
	8			8	8
	9			9	9
	10				
	11				

16. **HOW MANY YEARS OF SCHOOL HAVE YOU FINISHED?** ***(Mark the highest grade completed)***

- ○ Did not complete 6th grade
- ○ 6th - 8th grade
- ○ 9th - 10th grade
- ○ 11th - 12th grade
- ○ Vocational school
- ○ Some college
- ○ Graduated college
- ○ Graduate or professional school

17. **HAVE YOU EVER SMOKED A TOTAL OF 20 OR MORE PACKS OF CIGARETTES IN YOUR LIFETIME?**

- ○ No *(go to question 18)*
- ○ Yes, and I currently smoke
- ○ Yes, but I quit smoking

IF YES, WHAT IS THE TOTAL NUMBER OF YEARS YOU SMOKED?

- ○ 10 years or less
- ○ 11 - 20 years
- ○ 21 - 30 years
- ○ 31 - 40 years
- ○ 41 years or more

WHAT IS THE AVERAGE NUMBER OF CIGARETTES THAT YOU SMOKED *PER* DAY?

- ○ 5 cigarettes or less
- ○ 6 - 10 cigarettes
- ○ 11 - 20 cigarettes
- ○ 21 - 30 cigarettes
- ○ 31 cigarettes or more

IF YOU QUIT SMOKING, HOW LONG AGO DID YOU QUIT?

- ○ Less than 1 year
- ○ 1 - 2 years
- ○ 3 - 5 years
- ○ 6 - 10 years
- ○ 11 - 15 years
- ○ 16 - 20 years
- ○ 21 years or more

18. **WHAT WAS THE _NATURAL_ COLOR OF YOUR HAIR AT _AGE 20_?**

- ○ Black
- ○ Blonde
- ○ Light brown
- ○ Medium or dark brown
- ○ Red

19. **WHAT IS THE COLOR OF YOUR EYES?**

- ○ Brown or black
- ○ Blue
- ○ Gray
- ○ Green

20. **IF YOU HAD TO BE IN THE HOT SUN _WITHOUT PROTECTION_ FOR ONE HOUR, YOU WOULD GET A. . .**

- ○ Severe burn with blistering
- ○ Severe burn without blistering
- ○ Mild burn, then tan or darken
- ○ No burn, but would tan or darken
- ○ No burn and no tan

21. **IF YOU HAD TO BE IN THE SUN REPEATEDLY, YOUR SKIN WOULD TAN OR DARKEN. . .**

- ○ Deeply
- ○ Moderately
- ○ Lightly
- ○ Not at all

22. **HAVE YOU EVER BEEN SUNBURNED SEVERELY ENOUGH TO CAUSE BLISTERING?**

- ○ No *(go to question 23)*
- ○ Yes

IF YES, AT WHAT AGE DID THIS FIRST OCCUR?

- ○ 0 - 5 years
- ○ 6 - 10 years
- ○ 11 - 15 years
- ○ 16 - 20 years
- ○ 21 - 25 years
- ○ 26 years or older

HOW MANY TIMES HAS THIS OCCURRED?

- ○ 0 - 5 times
- ○ 6 - 10 times
- ○ 11 - 15 times
- ○ 16 - 20 times
- ○ 21 - 25 times
- ○ 26 times or more

23. **HAS YOUR DOCTOR EVER TOLD YOU THAT YOU HAD ANY OF THE FOLLOWING?** ***(Mark all that apply)***

- ○ High blood pressure
- ○ Heart attack or angina (chest pain on exertion that is relieved by medication)
- ○ Stroke
- ○ Diabetes (high blood sugar)
- ○ Tuberculosis (TB)
- ○ Gout (high uric acid)
- ○ Polyps of intestines
- ○ Ulcer (stomach or duodenal)
- ○ Partial removal of stomach
- ○ Kidney stones
- ○ Gallstones
- ○ Gallbladder removed
- ○ Blood transfusions
- ○ Asthma, hayfever, skin allergy, food allergy or any other allergy
- ○ Glaucoma
- ○ Cataract surgery
- ○ Colon or rectal cancer
- ○ Stomach cancer
- ○ Melanoma
- ○ Other skin cancer
- ○ Breast cancer
- ○ Prostate cancer (men only)
- ○ Cervix cancer (women only)
- ○ Other uterine cancer (women only)
- ○ Other cancer *(fill in circle and write in kind):* ________
- ○ **None of the above**

MEN ONLY

- ○ Vasectomy
- ○ Enlarged prostate
- ○ Surgery for enlarged prostate

EATING HABITS

24. The next questions are about your usual eating habits DURING THE LAST YEAR. For each food group, please fill in the circle that best describes HOW OFTEN you ate those items and then fill in the circle that best describes your USUAL SERVING SIZE.

Most categories include examples. They are only suggestions, and you may not eat all of the listed items. Some ethnic foods are also listed. If you don't recognize the name, you probably don't eat that item.

For each item, please include any fresh, frozen, canned, and packaged foods you ate, such as TV dinners, frozen entrees, vegetables, or side dishes.

If you did not eat an item, or if you ate an item less than once a month, fill in the circle in the first column. DO NOT LEAVE BLANK. It is not necessary to choose a serving size for these items.

For some categories, pictures of food on a dinner plate are included to help you estimate your usual serving size. Please note that "1 cup" refers to an 8-ounce (240 ml.) measuring cup.

For EACH FOOD GROUP, fill in the circle ○ that best describes HOW OFTEN you ate those items during the last year. Then fill in the circle ○ that best describes your USUAL SERVING SIZE.

SOUPS, RAMEN, AND JOOK	AVERAGE USE DURING LAST YEAR								YOUR USUAL SERVING SIZE
	Never or hardly ever	Once a month	2 to 3 times a month	Once a week	2 to 3 times a week	4 to 6 times a week	Once a day	2 or more times a day	
**Cream Soup or Chowder**	○	○	○	○	○	○	○	○	CHOOSE ONE<br>○ 1/2 cup or less **OR**<br>○ Small bowl (about 1 cup) **OR**<br>○ Large bowl (2 cups or more)
**Dried Bean or Pea (Legume) Soup** (such as Portuguese bean, split pea)	○	○	○	○	○	○	○	○	CHOOSE ONE<br>○ 1/2 cup or less **OR**<br>○ Small bowl (about 1 cup) **OR**<br>○ Large bowl (2 cups or more)
**Tomato or Vegetable Soup** (may include meat, poultry, or fish)	○	○	○	○	○	○	○	○	CHOOSE ONE<br>○ 1/2 cup or less **OR**<br>○ Small bowl (about 1 cup) **OR**<br>○ Large bowl (2 cups or more)
**Miso Soup**	○	○	○	○	○	○	○	○	CHOOSE ONE<br>○ 1/2 cup or less **OR**<br>○ Small bowl (about 1 cup) **OR**<br>○ Large bowl (2 cups or more)
**Broth with Noodles or Rice** (such as beef noodle, chicken rice, won tun mein)	○	○	○	○	○	○	○	○	CHOOSE ONE<br>○ 1/2 cup or less **OR**<br>○ Small bowl (about 1 cup) **OR**<br>○ Large bowl (2 cups or more)
**Mexican Meat Soup or Stew** (such as menudo, albondigas, cocido, pozole)	○	○	○	○	○	○	○	○	CHOOSE ONE<br>○ 1/2 cup or less **OR**<br>○ Small bowl (about 1 cup) **OR**<br>○ Large bowl (2 cups or more)
**Ramen or Saimin** (Oriental noodles with broth)	○	○	○	○	○	○	○	○	CHOOSE ONE<br>○ 1/2 cup or less **OR**<br>○ Small bowl (about 1 cup) **OR**<br>○ Large bowl (2 cups or more)
**Jook** (rice gruel - may include meat, poultry, fish, or vegetables)	○	○	○	○	○	○	○	○	CHOOSE ONE<br>○ 1/2 cup or less **OR**<br>○ Small bowl (about 1 cup) **OR**<br>○ Large bowl (2 cups or more)

For EACH FOOD GROUP, fill in the circle ○ that best describes HOW OFTEN you ate those items during the last year. Then fill in the circle ○ that best describes your USUAL SERVING SIZE.

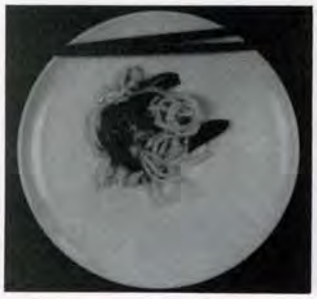

A

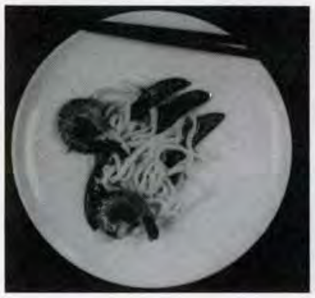

B

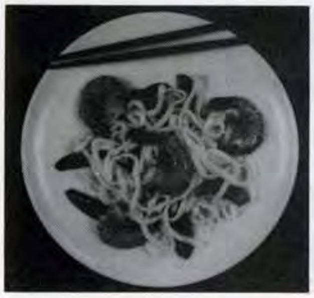

C

NOODLES, SPAGHETTI, AND MIXED DISHES	AVERAGE USE DURING LAST YEAR								YOUR USUAL SERVING SIZE
	Never or hardly ever	Once a month	2 to 3 times a month	Once a week	2 to 3 times a week	4 to 6 times a week	Once a day	2 or more times a day	
**Chow Mein, Chow Fun, or Yakisoba** (Oriental fried noodles)	○	○	○	○	○	○	○	○	**CHOOSE ONE**<br>○ Photo A (1/2 cup or less) **OR**<br>○ Photo B (about 1 cup) **OR**<br>○ Photo C (2 cups or more)
**Spaghetti, Ravioli, Lasagna, or Other Pasta with Tomato Sauce**	○	○	○	○	○	○	○	○	**CHOOSE ONE**<br>○ Photo A (1/2 cup or less) **OR**<br>○ Photo B (about 1 cup) **OR**<br>○ Photo C (2 cups or more)
**Macaroni and Cheese or Other Pasta and Cheese Casseroles**	○	○	○	○	○	○	○	○	**CHOOSE ONE**<br>○ Photo A (1/2 cup or less) **OR**<br>○ Photo B (about 1 cup) **OR**<br>○ Photo C (2 cups or more)
**Macaroni or Potato Salad** (with mayonnaise)	○	○	○	○	○	○	○	○	**CHOOSE ONE**<br>○ Photo A (1/2 cup or less) **OR**<br>○ Photo B (about 1 cup) **OR**<br>○ Photo C (2 cups or more)
**Pasta or Somen Salad**	○	○	○	○	○	○	○	○	**CHOOSE ONE**<br>○ Photo A (1/2 cup or less) **OR**<br>○ Photo B (about 1 cup) **OR**<br>○ Photo C (2 cups or more)
**Noodle Casseroles** (with tuna, chicken or turkey)	○	○	○	○	○	○	○	○	**CHOOSE ONE**<br>○ Photo A (1/2 cup or less) **OR**<br>○ Photo B (about 1 cup) **OR**<br>○ Photo C (2 cups or more)
**Pasta with Cream Sauce** (such as linguine with clam sauce, beef stroganoff)	○	○	○	○	○	○	○	○	**CHOOSE ONE**<br>○ Photo A (1/2 cup or less) **OR**<br>○ Photo B (about 1 cup) **OR**<br>○ Photo C (2 cups or more)
**Arroz Con Pollo** (rice with chicken)	○	○	○	○	○	○	○	○	**CHOOSE ONE**<br>○ Photo A (1/2 cup or less) **OR**<br>○ Photo B (about 1 cup) **OR**<br>○ Photo C (2 cups or more)
**Stew, Curry, Pot Pie or Empanada** (with beef or lamb)	○	○	○	○	○	○	○	○	**CHOOSE ONE**<br>○ Photo A (1/2 cup or 1 Empanada) **OR**<br>○ Photo B (about 1 cup or 1 pie) **OR**<br>○ Photo C (2 cups or more)
**Stew, Curry, Pot Pie or Empanada** (with chicken or turkey)	○	○	○	○	○	○	○	○	**CHOOSE ONE**<br>○ Photo A (1/2 cup or 1 Empanada) **OR**<br>○ Photo B (about 1 cup or 1 pie) **OR**<br>○ Photo C (2 cups or more)

For EACH FOOD GROUP, fill in the circle ○ that best describes HOW OFTEN you ate those items during the last year. Then fill in the circle ○ that best describes your USUAL SERVING SIZE.

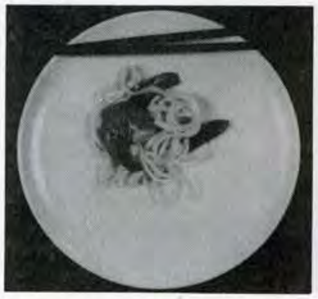

A

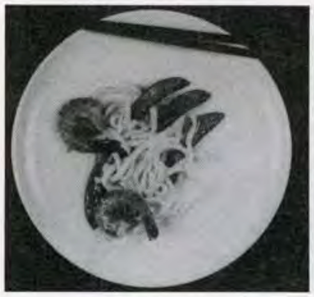

B

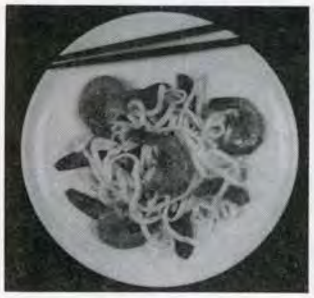

C

**MIXED DISHES**	**AVERAGE USE DURING LAST YEAR**								**YOUR USUAL SERVING SIZE**
	**Never or hardly ever**	**Once a month**	**2 to 3 times a month**	**Once a week**	**2 to 3 times a week**	**4 to 6 times a week**	**Once a day**	**2 or more times a day**	
**Stir-Fried Beef or Pork and Vegetables, or Fajitas** (such as beef broccoli, pork tofu, chop suey, sukiyaki)	○	○	○	○	○	○	○	○	**CHOOSE ONE**<br>○ Photo A (1/2 cup or less) **OR**<br>○ Photo B (about 1 cup) **OR**<br>○ Photo C (2 cups or more)
**Stir-Fried Chicken and Vegetables, or Fajitas** (such as sukiyaki, nishime, chicken long rice)	○	○	○	○	○	○	○	○	**CHOOSE ONE**<br>○ Photo A (1/2 cup or less) **OR**<br>○ Photo B (about 1 cup) **OR**<br>○ Photo C (2 cups or more)
**Stir-Fried Shrimp or Fish and Vegetables**	○	○	○	○	○	○	○	○	**CHOOSE ONE**<br>○ Photo A (1/2 cup or less) **OR**<br>○ Photo B (about 1 cup) **OR**<br>○ Photo C (2 cups or more)
**Stir-Fried Vegetables** (no meat)	○	○	○	○	○	○	○	○	**CHOOSE ONE**<br>○ Photo A (1/2 cup or less) **OR**<br>○ Photo B (about 1 cup) **OR**<br>○ Photo C (2 cups or more)
**Pork and Greens or Laulaus**	○	○	○	○	○	○	○	○	**CHOOSE ONE**<br>○ Photo A (1/2 cup or less) **OR**<br>○ Photo B or 1 laulau **OR**<br>○ Photo C or 2 laulaus or more
**Chili**	○	○	○	○	○	○	○	○	**CHOOSE ONE**<br>○ 1/2 cup or less **OR**<br>○ Small bowl (1 cup) **OR**<br>○ Large bowl (2 cups or more)
**Hamburgers** (on a bun)	○	○	○	○	○	○	○	○	**CHOOSE ONE**<br>○ 1 regular size burger **OR**<br>○ 1 quarter-pound burger **OR**<br>○ 1 large double burger
**Cheeseburgers** (on a bun)	○	○	○	○	○	○	○	○	**CHOOSE ONE**<br>○ 1 regular size burger **OR**<br>○ 1 quarter-pound burger **OR**<br>○ 1 large double burger
**Meat Loaf, Meatballs, or Patties** (not fast-food hamburgers)	○	○	○	○	○	○	○	○	**CHOOSE ONE**<br>○ 1 to 2 meatballs **OR**<br>○ 1 patty or slice or 3 meatballs **OR**<br>○ 1 large patty or 5 meatballs
**Pizza**	○	○	○	○	○	○	○	○	**CHOOSE ONE**<br>○ 1 piece or slice or less **OR**<br>○ 2 to 3 pieces **OR**<br>○ 4 pieces or more

For EACH FOOD GROUP, fill in the circle ○ that best describes HOW OFTEN you ate those items during the last year. Then fill in the circle ○ that best describes your USUAL SERVING SIZE.

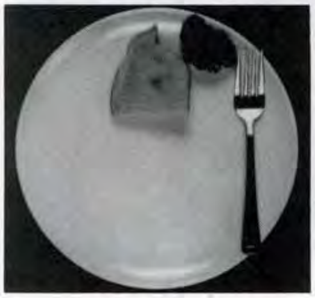

A

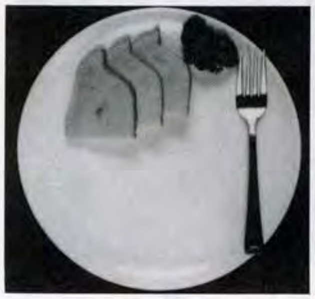

B

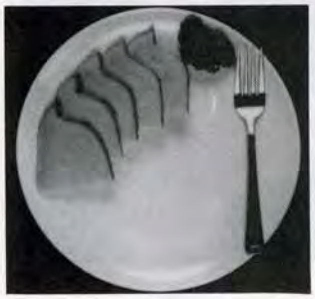

C

**MEATS (NOT PART OF MIXED DISHES)**	**AVERAGE USE DURING LAST YEAR**								**YOUR USUAL SERVING SIZE**
	**Never or hardly ever**	**Once a month**	**2 to 3 times a month**	**Once a week**	**2 to 3 times a week**	**4 to 6 times a week**	**Once a day**	**2 or more times a day**	
**Beef Steak or Roast, Veal or Lamb** (includes beef teriyaki, chile colorado and carne asada)	○	○	○	○	○	○	○	○	**CHOOSE ONE**<br>○ Photo A (1 ounce or less) **OR**<br>○ Photo B (3 oz. or 1 lamb chop) **OR**<br>○ Photo C (5 ounces or more)
**Shortribs**	○	○	○	○	○	○	○	○	**CHOOSE ONE**<br>○ Photo A (1 ounce or less) **OR**<br>○ Photo B (or 2 shortribs) **OR**<br>○ Photo C (or 3 ribs or more)
**Corned Beef** (fresh or canned)	○	○	○	○	○	○	○	○	**CHOOSE ONE**<br>○ Photo A (1 ounce or less) **OR**<br>○ Photo B (or 1/4 12-oz. tin) **OR**<br>○ Photo C (or 1/2 12-oz. tin or more)
**Corned Beef Hash**	○	○	○	○	○	○	○	○	**CHOOSE ONE**<br>○ Photo A or 1 patty **OR**<br>○ Photo B or 2 patties **OR**<br>○ Photo C or 3 patties or more
**Pork Chops or Roasts, Kalua Pig, or Carnitas** (includes chile verde)	○	○	○	○	○	○	○	○	**CHOOSE ONE**<br>○ Photo A (1 ounce or less) **OR**<br>○ Photo B (3 ounces) **OR**<br>○ Photo C (5 ounces or more)
**Ham** (includes baked, fried, or sandwich)	○	○	○	○	○	○	○	○	**CHOOSE ONE**<br>○ Photo A (1 ounce or less) **OR**<br>○ Photo B (3 ounces) **OR**<br>○ Photo C (5 ounces or more)
**Ham Hocks or Pig's Feet**	○	○	○	○	○	○	○	○	**CHOOSE ONE**<br>○ Photo A (1 ounce or less) **OR**<br>○ Photo B (3 ounces) **OR**<br>○ Photo C (5 ounces or more)
**Spareribs**	○	○	○	○	○	○	○	○	**CHOOSE ONE**<br>○ 3 small or 1 long rib or less **OR**<br>○ 2 to 3 long ribs (5-7 inches) **OR**<br>○ 4 long ribs or more
**Liver**	○	○	○	○	○	○	○	○	**CHOOSE ONE**<br>○ Photo A (1 ounce or less) **OR**<br>○ Photo B or 3 chicken livers **OR**<br>○ Photo C (5 ounces or more)
**Chicken or Turkey Wings**	○	○	○	○	○	○	○	○	**CHOOSE ONE**<br>○ 2 chicken wings or less **OR**<br>○ 3 chicken wings **OR**<br>○ 1 turkey or 4 chicken wings or more

For EACH FOOD GROUP, fill in the circle ○ that best describes HOW OFTEN you ate those items during the last year. Then fill in the circle ○ that best describes your USUAL SERVING SIZE.

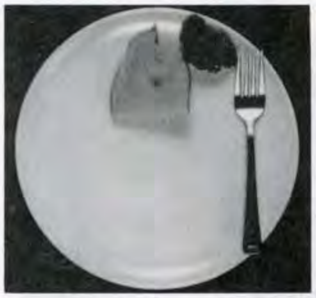

A

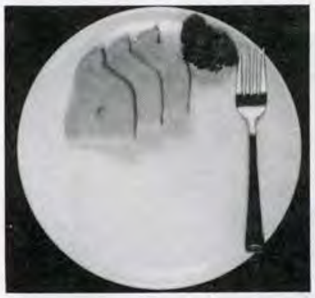

B

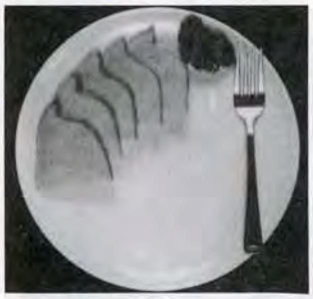

C

**POULTRY AND FISH (NOT PART OF MIXED DISHES)**	**AVERAGE USE DURING LAST YEAR**								**YOUR USUAL SERVING SIZE**
	**Never or hardly ever**	**Once a month**	**2 to 3 times a month**	**Once a week**	**2 to 3 times a week**	**4 to 6 times a week**	**Once a day**	**2 or more times a day**	
**Fried Chicken** (includes fried chicken sandwich, nuggets)	○	○	○	○	○	○	○	○	**CHOOSE ONE**<br>○ Photo A (or 1 drumstick) **OR**<br>○ Photo B (or 1 breast, 2 thighs, 3 wings, or 1 sandwich) **OR**<br>○ Photo C (or 2 breasts or 4 thighs)
**Roasted, Baked, Grilled or Stewed Chicken** (includes grilled chicken sandwich)	○	○	○	○	○	○	○	○	**CHOOSE ONE**<br>○ Photo A (or 1 drumstick) **OR**<br>○ Photo B (or 1 breast, 2 thighs, 3 wings, or 1 sandwich) **OR**<br>○ Photo C (or 2 breasts or 4 thighs)
**Turkey** (includes roast, ground, deli-style, or sandwich)	○	○	○	○	○	○	○	○	**CHOOSE ONE**<br>○ Photo A (1 ounce or less) **OR**<br>○ Photo B (3 ounces) **OR**<br>○ Photo C (5 ounces or more)
**Fried Shrimp or Other Shellfish** (includes tempura, fried calamari or squid)	○	○	○	○	○	○	○	○	**CHOOSE ONE**<br>○ 1 to 3 items **OR**<br>○ 4 to 5 items or 1/2 cup **OR**<br>○ 6 items or more
**Cooked, Canned, or Raw Shellfish** (such as crab, squid, shrimp)	○	○	○	○	○	○	○	○	**CHOOSE ONE**<br>○ 5-6 shrimp or 1/4 cup **OR**<br>○ 1 crab or 1/2 cup **OR**<br>○ 1 lobster tail or 1 cup or more
**Fried Fish** (includes pan-fried fish, frozen fish sticks, fried fish sandwich)	○	○	○	○	○	○	○	○	**CHOOSE ONE**<br>○ Photo A (about 1 ounce) **OR**<br>○ Photo B (3 oz. or 1 sandwich) **OR**<br>○ Photo C (5 ounces or more)
**Baked, Broiled, Boiled or Raw Fish** (such as red snapper, salmon, sashimi)	○	○	○	○	○	○	○	○	**CHOOSE ONE**<br>○ Photo A (about 1 ounce) **OR**<br>○ Photo B (3 ounces) **OR**<br>○ Photo C (5 ounces or more)
**Canned Tunafish** (plain, salad, or sandwich)	○	○	○	○	○	○	○	○	**CHOOSE ONE**<br>○ 1/4 cup or 1/2 sandwich **OR**<br>○ 1/2 cup or 1 sandwich **OR**<br>○ 1 cup or 2 sandwiches
**Other Canned Fish** (such as salmon, mackerel, sardines)	○	○	○	○	○	○	○	○	**CHOOSE ONE**<br>○ 3 small sardines or 1/4 cup **OR**<br>○ 1/2 cup fish **OR**<br>○ 1 cup fish or more
**Salted and Dried Fish** (such as ike, cuttlefish, iriko)	○	○	○	○	○	○	○	○	**CHOOSE ONE**<br>○ 1 slice or strip or piece **OR**<br>○ 2 slices **OR**<br>○ 4 slices or more

PROCESSED MEATS AND MEXICAN DISHES	AVERAGE USE DURING LAST YEAR								YOUR USUAL SERVING SIZE
	Never or hardly ever	Once a month	2 to 3 times a month	Once a week	2 to 3 times a week	4 to 6 times a week	Once a day	2 or more times a day	
**Bacon** (includes Canadian bacon)	◯	◯	◯	◯	◯	◯	◯	◯	**CHOOSE ONE** ◯ 1 slice or strip or piece **OR** ◯ 2 slices **OR** ◯ 3 slices or more
**Regular Hot Dogs** (beef or pork)	◯	◯	◯	◯	◯	◯	◯	◯	**CHOOSE ONE** ◯ 1/2 hot dog **OR** ◯ 1 hot dog **OR** ◯ 2 hot dogs or more
**Chicken or Turkey Hot Dogs or Luncheon Meats**	◯	◯	◯	◯	◯	◯	◯	◯	**CHOOSE ONE** ◯ 1/2 hot dog or 1 slice **OR** ◯ 1 hot dog or 2 slices **OR** ◯ 2 hot dogs or 3 slices or more
**Spam, Bologna, Salami, Pastrami or Other Luncheon Meats**	◯	◯	◯	◯	◯	◯	◯	◯	**CHOOSE ONE** ◯ 1 slice (1 ounce or less) **OR** ◯ 2 slices **OR** ◯ 3 slices or more
**Sausage** (such as pork, beef, chorizo, Polish, Vienna, Portuguese, hot links)	◯	◯	◯	◯	◯	◯	◯	◯	**CHOOSE ONE** ◯ 1 piece or link **OR** ◯ 2-3 pieces or links or 1 patty **OR** ◯ 4 pieces or links or more
**Tacos, Tostadas, Sopes, or Taco Salad** (with beef or pork)	◯	◯	◯	◯	◯	◯	◯	◯	**CHOOSE ONE** ◯ 1 item or less **OR** ◯ 2 items **OR** ◯ 3 items or more
**Tacos, Tostadas, Sopes, or Taco Salad** (with chicken)	◯	◯	◯	◯	◯	◯	◯	◯	**CHOOSE ONE** ◯ 1 item or less **OR** ◯ 2 items **OR** ◯ 3 items or more
**Meat Burritos** (includes beef and bean and other combinations)	◯	◯	◯	◯	◯	◯	◯	◯	**CHOOSE ONE** ◯ 1 fast-food burrito **OR** ◯ 1 medium burrito **OR** ◯ 1 large or 2 fast-food burritos
**Vegetable or Bean Burritos, Tacos, or Tostadas** (no meat)	◯	◯	◯	◯	◯	◯	◯	◯	**CHOOSE ONE** ◯ 1 item or less **OR** ◯ 2 items **OR** ◯ 3 items or more
**Enchiladas with Chicken**	◯	◯	◯	◯	◯	◯	◯	◯	**CHOOSE ONE** ◯ 1 enchilada or less **OR** ◯ 2 enchiladas **OR** ◯ 3 enchiladas or more
**Enchiladas with Beef**	◯	◯	◯	◯	◯	◯	◯	◯	**CHOOSE ONE** ◯ 1 enchilada or less **OR** ◯ 2 enchiladas **OR** ◯ 3 enchiladas or more
**Enchiladas with Cheese, Quesadillas, or Nachos with Cheese**	◯	◯	◯	◯	◯	◯	◯	◯	**CHOOSE ONE** ◯ 1 enchilada or small quesadilla **OR** ◯ 2 enchiladas or 1 serving nachos **OR** ◯ 3 enchiladas
**Tamales**	◯	◯	◯	◯	◯	◯	◯	◯	**CHOOSE ONE** ◯ 1/2 tamale or less **OR** ◯ 1 tamale **OR** ◯ 2 tamales or more
**Chili Rellenos**	◯	◯	◯	◯	◯	◯	◯	◯	**CHOOSE ONE** ◯ 1/2 chili relleno or less **OR** ◯ 1 chili relleno **OR** ◯ 2 chili rellenos or more

For EACH FOOD GROUP, fill in the circle ◯ that best describes HOW OFTEN you ate those items during the last year. Then fill in the circle ◯ that best describes your USUAL SERVING SIZE.

RICE, POTATOES, TARO, AND POI	AVERAGE USE DURING LAST YEAR: Never or hardly ever	Once a month	2 to 3 times a month	Once a week	2 to 3 times a week	4 to 6 times a week	Once a day	2 or more times a day	YOUR USUAL SERVING SIZE
**White Rice** (includes musubi)	◯	◯	◯	◯	◯	◯	◯	◯	**CHOOSE ONE**<br>◯ 1/2 cup or 1 scoop or less **OR**<br>◯ 1 rice bowl (1 cup) or 1 musubi **OR**<br>◯ 2 rice bowls or 2 musubi or more
**Sushi or Barazushi**	◯	◯	◯	◯	◯	◯	◯	◯	**CHOOSE ONE**<br>◯ 1-2 pieces or small cone **OR**<br>◯ 3-4 pieces or 1 large cone or 1/2 cup **OR**<br>◯ 5 pieces or 1 cup or more
**Brown or Wild Rice**	◯	◯	◯	◯	◯	◯	◯	◯	**CHOOSE ONE**<br>◯ 1/2 cup or 1 scoop or less **OR**<br>◯ 1 cup or 2 scoops **OR**<br>◯ 2 cups or more
**Mexican or Spanish Rice**	◯	◯	◯	◯	◯	◯	◯	◯	**CHOOSE ONE**<br>◯ 1/2 cup or less **OR**<br>◯ 1 cup **OR**<br>◯ 2 cups or more
**Fried Rice**	◯	◯	◯	◯	◯	◯	◯	◯	**CHOOSE ONE**<br>◯ 1/2 cup or less **OR**<br>◯ 1 cup **OR**<br>◯ 2 cups or more
**French-Fried, Hash-Browned or other Fried Potatoes**	◯	◯	◯	◯	◯	◯	◯	◯	**CHOOSE ONE**<br>◯ fast-food small order or 1 cup **OR**<br>◯ fast-food medium order **OR**<br>◯ fast-food large order or more
**Mashed, Scalloped or Au Gratin Potatoes**	◯	◯	◯	◯	◯	◯	◯	◯	**CHOOSE ONE**<br>◯ 1/2 cup or 1 scoop or less **OR**<br>◯ 1 cup or 2 scoops **OR**<br>◯ 2 cups or more
**Baked or Boiled White Potatoes**	◯	◯	◯	◯	◯	◯	◯	◯	**CHOOSE ONE**<br>◯ 1 small or 1/2 medium or less **OR**<br>◯ 1 medium (about 5 inches) **OR**<br>◯ 1 large potato or more
**Yellow-Orange Sweet Potatoes or Yams**	◯	◯	◯	◯	◯	◯	◯	◯	**CHOOSE ONE**<br>◯ 1 small or 1/2 medium or less **OR**<br>◯ 1 medium (about 5 inches) **OR**<br>◯ 1 large potato or more
**White or Purple Sweet Potatoes**	◯	◯	◯	◯	◯	◯	◯	◯	**CHOOSE ONE**<br>◯ 1 small or 1/2 medium or less **OR**<br>◯ 1 medium (about 5 inches) **OR**<br>◯ 1 large potato or more
**Taro**	◯	◯	◯	◯	◯	◯	◯	◯	**CHOOSE ONE**<br>◯ 1/4 taro or less **OR**<br>◯ 1/2 taro **OR**<br>◯ 1 whole taro or more
**Poi**	◯	◯	◯	◯	◯	◯	◯	◯	**CHOOSE ONE**<br>◯ 1/4 cup or less **OR**<br>◯ 1/2 cup **OR**<br>◯ 1 cup or more

For EACH FOOD GROUP, fill in the circle ◯ that best describes HOW OFTEN you ate those items during the last year. Then fill in the circle ◯ that best describes your USUAL SERVING SIZE.

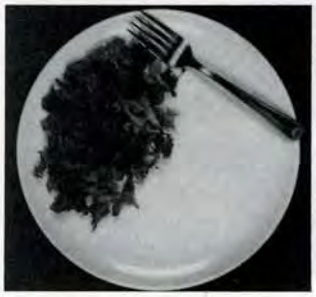

A

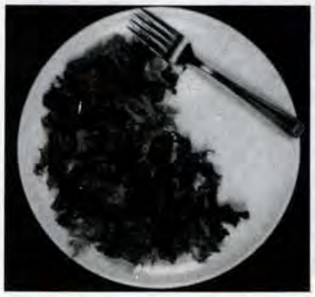

B

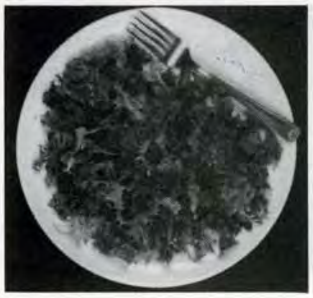

C

SALAD ITEMS, EGGS, AND OTHER NON-MEAT ITEMS	AVERAGE USE DURING LAST YEAR								YOUR USUAL SERVING SIZE
	Never or hardly ever	Once a month	2 to 3 times a month	Once a week	2 to 3 times a week	4 to 6 times a week	Once a day	2 or more times a day	
**Light Green Lettuce or Tossed Salad** (such as iceberg or head lettuce)	◯	◯	◯	◯	◯	◯	◯	◯	**CHOOSE ONE**<br>◯ Photo A (1/2 cup or less) **OR**<br>◯ Photo B (about 1 cup) **OR**<br>◯ Photo C (1-1/2 cups or more)
**Dark Green Lettuce** (such as romaine, red, butter, manoa, endive)	◯	◯	◯	◯	◯	◯	◯	◯	**CHOOSE ONE**<br>◯ Photo A (1/2 cup or less) **OR**<br>◯ Photo B (about 1 cup) **OR**<br>◯ Photo C (1-1/2 cups or more)
**Tomatoes**	◯	◯	◯	◯	◯	◯	◯	◯	**CHOOSE ONE**<br>◯ 2 slices or wedges or 2 cherry tomatoes or less **OR**<br>◯ 4 slices or 1/2 medium tomato **OR**<br>◯ 1 medium tomato or more
**Coleslaw**	◯	◯	◯	◯	◯	◯	◯	◯	**CHOOSE ONE**<br>◯ 1/4 cup or less **OR**<br>◯ 1/2 cup **OR**<br>◯ 1 cup or more
**Regular Salad Dressings or Mayonnaise Added to Salads**	◯	◯	◯	◯	◯	◯	◯	◯	**CHOOSE ONE**<br>◯ 2 teaspoons or less **OR**<br>◯ 1 Tablespoon **OR**<br>◯ 2 Tablespoons or more
**Low-Calorie or Diet Dressings Added to Salads**	◯	◯	◯	◯	◯	◯	◯	◯	**CHOOSE ONE**<br>◯ 2 teaspoons or less **OR**<br>◯ 1 Tablespoon **OR**<br>◯ 2 Tablespoons or more
**Eggs, Cooked or Raw** (includes egg salad)	◯	◯	◯	◯	◯	◯	◯	◯	**CHOOSE ONE**<br>◯ 1/2 egg **OR**<br>◯ 1 egg or 1 sandwich **OR**<br>◯ 2 eggs or more
**Egg Substitute**	◯	◯	◯	◯	◯	◯	◯	◯	**CHOOSE ONE**<br>◯ 2 Tablespoons **OR**<br>◯ 1/4 cup (= 1 egg) **OR**<br>◯ 1/2 cup (= 2 eggs) or more
**Tofu** (soybean curd)	◯	◯	◯	◯	◯	◯	◯	◯	**CHOOSE ONE**<br>◯ 2 cubes or 1/4 cup **OR**<br>◯ 1/4 block or 1/2 cup **OR**<br>◯ 1/2 block or more
**Vegetarian Meat Loaf, Meatballs or Patties**	◯	◯	◯	◯	◯	◯	◯	◯	**CHOOSE ONE**<br>◯ 1 to 2 meatballs **OR**<br>◯ 1 patty or slice or 3 meatballs **OR**<br>◯ 1 large patty, 5 meatballs or more

For EACH FOOD GROUP, fill in the circle ◯ that best describes HOW OFTEN you ate those items during the last year. Then fill in the circle ◯ that best describes your USUAL SERVING SIZE.

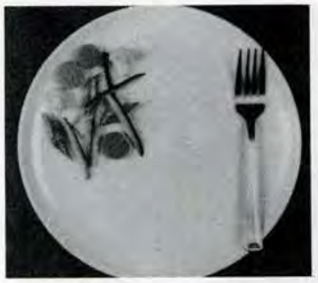

A

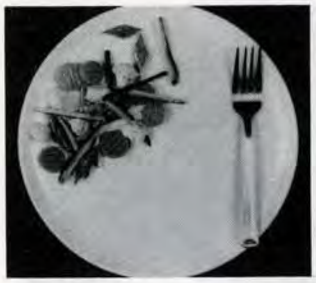

B

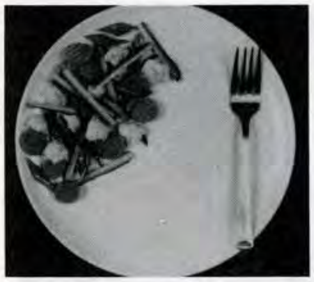

C

RAW OR COOKED VEGETABLES (NOT IN SOUPS OR MIXED DISHES)	AVERAGE USE DURING LAST YEAR								YOUR USUAL SERVING SIZE
	Never or hardly ever	Once a month	2 to 3 times a month	Once a week	2 to 3 times a week	4 to 6 times a week	Once a day	2 or more times a day	
**Broccoli** (raw or cooked)	◯	◯	◯	◯	◯	◯	◯	◯	CHOOSE ONE<br>◯ Photo A (1/4 cup or less) **OR**<br>◯ Photo B (about 1/2 cup) **OR**<br>◯ Photo C (1 cup or more)
**Cabbage** (such as head, Chinese or Napa cabbage, Brussels sprouts)	◯	◯	◯	◯	◯	◯	◯	◯	CHOOSE ONE<br>◯ Photo A (1/4 cup or less) **OR**<br>◯ Photo B (about 1/2 cup) **OR**<br>◯ Photo C (1 cup or more)
**Dark Leafy Greens** (such as spinach, collard, mustard or turnip greens, bok choy, watercress, chard)	◯	◯	◯	◯	◯	◯	◯	◯	CHOOSE ONE<br>◯ Photo A (1/4 cup or less) **OR**<br>◯ Photo B (about 1/2 cup) **OR**<br>◯ Photo C (1 cup or more)
**Green Beans or Peas**	◯	◯	◯	◯	◯	◯	◯	◯	CHOOSE ONE<br>◯ Photo A (1/4 cup or less) **OR**<br>◯ Photo B (about 1/2 cup) **OR**<br>◯ Photo C (1 cup or more)
**Other Green Vegetables** (such as zucchini, celery, asparagus, green pepper, okra)	◯	◯	◯	◯	◯	◯	◯	◯	CHOOSE ONE<br>◯ Photo A (1/4 cup or less) **OR**<br>◯ Photo B (about 1/2 cup) **OR**<br>◯ Photo C (1 cup or more)
**Cauliflower**	◯	◯	◯	◯	◯	◯	◯	◯	CHOOSE ONE<br>◯ Photo A (1/4 cup or less) **OR**<br>◯ Photo B (about 1/2 cup) **OR**<br>◯ Photo C (1 cup or more)
**Carrots** (raw or cooked)	◯	◯	◯	◯	◯	◯	◯	◯	CHOOSE ONE<br>◯ Photo A (or 4-5 sticks or less) **OR**<br>◯ Photo B (1/2 cup or 1 med.) **OR**<br>◯ Photo C (1 cup or more)
**Corn** (fresh, frozen, or canned)	◯	◯	◯	◯	◯	◯	◯	◯	CHOOSE ONE<br>◯ Photo A (1/4 cup or less) **OR**<br>◯ Photo B (1/2 cup or 1 cob) **OR**<br>◯ Photo C (1 cup or more)
**Pumpkin or Yellow-Orange Winter Squash**	◯	◯	◯	◯	◯	◯	◯	◯	CHOOSE ONE<br>◯ Photo A (1/4 cup or less) **OR**<br>◯ Photo B (about 1/2 cup) **OR**<br>◯ Photo C (1 cup or more)
**Other Vegetables** (such as white or summer squash, beets, eggplant)	◯	◯	◯	◯	◯	◯	◯	◯	CHOOSE ONE<br>◯ Photo A (1/4 cup or less) **OR**<br>◯ Photo B (about 1/2 cup) **OR**<br>◯ Photo C (1 cup or more)

For EACH FOOD GROUP, fill in the circle ◯ that best describes HOW OFTEN you ate those items during the last year. Then fill in the circle ◯ that best describes your USUAL SERVING SIZE.

DRIED BEANS (NOT IN SOUPS OR MIXED DISHES)	AVERAGE USE DURING LAST YEAR								YOUR USUAL SERVING SIZE
	Never or hardly ever	Once a month	2 to 3 times a month	Once a week	2 to 3 times a week	4 to 6 times a week	Once a day	2 or more times a day	
**Refried Beans** (not in burritos or tostadas)	◯	◯	◯	◯	◯	◯	◯	◯	CHOOSE ONE<br>◯ Photo A (1/4 cup or less) **OR**<br>◯ Photo B (about 1/2 cup) **OR**<br>◯ Photo C (1 cup or more)
**Baked Beans or Pork and Beans**	◯	◯	◯	◯	◯	◯	◯	◯	CHOOSE ONE<br>◯ Photo A (1/4 cup or less) **OR**<br>◯ Photo B (about 1/2 cup) **OR**<br>◯ Photo C (1 cup or more)
**Boiled Dried Beans or Peas** (such as red, lima, pinto or soy beans, black-eyed peas, frijoles de la olla)	◯	◯	◯	◯	◯	◯	◯	◯	CHOOSE ONE<br>◯ Photo A (1/4 cup or less) **OR**<br>◯ Photo B (about 1/2 cup) **OR**<br>◯ Photo C (1 cup or more)

FRUITS AND JUICES	AVERAGE USE DURING LAST YEAR								YOUR USUAL SERVING SIZE
	Never or hardly ever	Once a month	2 to 3 times a month	Once a week	2 to 3 times a week	4 to 6 times a week	Once a day	2 or more times a day	
**Oranges**	◯	◯	◯	◯	◯	◯	◯	◯	CHOOSE ONE<br>◯ 1/2 orange or 1/2 cup or less **OR**<br>◯ 1 orange or 1 cup **OR**<br>◯ 2 oranges or more
**Tangerines or Mandarin Oranges**	◯	◯	◯	◯	◯	◯	◯	◯	CHOOSE ONE<br>◯ 1 tangerine or 1/2 cup or less **OR**<br>◯ 2 tangerines or 1 cup **OR**<br>◯ 3 tangerines or more
**Grapefruit or Pomelo**	◯	◯	◯	◯	◯	◯	◯	◯	CHOOSE ONE<br>◯ 1/4 cup or less **OR**<br>◯ 1/2 grapefruit or 1/2 cup **OR**<br>◯ 1 cup or more
**Papaya**	◯	◯	◯	◯	◯	◯	◯	◯	CHOOSE ONE<br>◯ 1/4 papaya or less **OR**<br>◯ 1/2 papaya **OR**<br>◯ 1 papaya or more
**Pineapple** (fresh or canned)	◯	◯	◯	◯	◯	◯	◯	◯	CHOOSE ONE<br>◯ 1 slice or wedge or less **OR**<br>◯ 1/2 cup or 2 slices or wedges **OR**<br>◯ 1 cup or more
**Peaches** (fresh, canned, or dried)	◯	◯	◯	◯	◯	◯	◯	◯	CHOOSE ONE<br>◯ 1/2 peach or less **OR**<br>◯ 1 peach or 2 halves or 1/2 cup **OR**<br>◯ 2 peaches or 1 cup or more
**Apricots** (fresh, canned, or dried)	◯	◯	◯	◯	◯	◯	◯	◯	CHOOSE ONE<br>◯ 1 apricot or less **OR**<br>◯ 2 apricots or 1/2 cup **OR**<br>◯ 3 apricots or more
**Pears** (fresh, canned, or dried)	◯	◯	◯	◯	◯	◯	◯	◯	CHOOSE ONE<br>◯ 1/2 pear or 1/2 cup **OR**<br>◯ 1 pear or 1 cup **OR**<br>◯ 2 pears or more

For EACH FOOD GROUP, fill in the circle ○ that best describes HOW OFTEN you ate those items during the last year. Then fill in the circle ○ that best describes your USUAL SERVING SIZE.

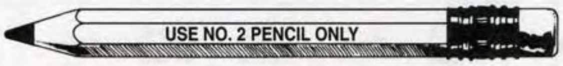

FRUITS AND JUICES (continued)	AVERAGE USE DURING LAST YEAR								YOUR USUAL SERVING SIZE
	Never or hardly ever	Once a month	2 to 3 times a month	Once a week	2 to 3 times a week	4 to 6 times a week	Once a day	2 or more times a day	
**Apples and Applesauce**	○	○	○	○	○	○	○	○	**CHOOSE ONE**<br>○ 1/2 apple or 1/2 cup **OR**<br>○ 1 apple or 1 cup **OR**<br>○ 2 apples or more
**Bananas**	○	○	○	○	○	○	○	○	**CHOOSE ONE**<br>○ 1/2 banana **OR**<br>○ 1 banana **OR**<br>○ 2 bananas or more
**Cantaloupe** (in season)	○	○	○	○	○	○	○	○	**CHOOSE ONE**<br>○ 1/4 cantaloupe or less **OR**<br>○ 1/2 cantaloupe **OR**<br>○ 1 cantaloupe or more
**Watermelon** (in season)	○	○	○	○	○	○	○	○	**CHOOSE ONE**<br>○ 1 quarter slice or 1/2 cup **OR**<br>○ 1 half slice or 1 cup **OR**<br>○ 1 whole slice or more
**Mangoes** (in season)	○	○	○	○	○	○	○	○	**CHOOSE ONE**<br>○ 1/2 cup slices **OR**<br>○ 1 medium or Pirie or 1 cup **OR**<br>○ 1 large or Hayden or more
**Avocados and Guacamole**	○	○	○	○	○	○	○	○	**CHOOSE ONE**<br>○ 2 slices or 2 Tablespoons **OR**<br>○ 1/4 avocado or 1/4 cup **OR**<br>○ 1/2 avocado or 1/2 cup or more
**Any Other Fruit** (fresh, canned, or dried)	○	○	○	○	○	○	○	○	**CHOOSE ONE**<br>○ 1/2 cup or less **OR**<br>○ 1 fruit or 1 cup **OR**<br>○ 2 fruits or more
**Orange or Grapefruit Juice** (not orange drinks or orange soda)	○	○	○	○	○	○	○	○	**CHOOSE ONE**<br>○ Small juice glass (1/2 cup) **OR**<br>○ Large glass (8 ounces) **OR**<br>○ 12-ounce can or more
**Tomato or V-8 Juice**	○	○	○	○	○	○	○	○	**CHOOSE ONE**<br>○ Small juice glass (1/2 cup) **OR**<br>○ Large glass (8 ounces) **OR**<br>○ 12-ounce can or more
**Other Fruit Juices or Fruit Drinks**	○	○	○	○	○	○	○	○	**CHOOSE ONE**<br>○ Small juice glass (1/2 cup) **OR**<br>○ Large glass (8 ounces) **OR**<br>○ 12-ounce can or more

For EACH FOOD GROUP, fill in the circle ◯ that best describes HOW OFTEN you ate those items during the last year. Then fill in the circle ◯ that best describes your USUAL SERVING SIZE.

BREAD ITEMS	AVERAGE USE DURING LAST YEAR: Never or hardly ever	Once a month	2 to 3 times a month	Once a week	2 to 3 times a week	4 to 6 times a week	Once a day	2 or more times a day	YOUR USUAL SERVING SIZE
**White Bread** (includes sandwich, French, sourdough, pan dulce, Portuguese sweet bread)	◯	◯	◯	◯	◯	◯	◯	◯	**CHOOSE ONE** ◯ 1 slice or less **OR** ◯ 2 slices **OR** ◯ 3 slices or more
**Whole Wheat or Rye Bread** (includes pumpernickel, whole wheat pita bread)	◯	◯	◯	◯	◯	◯	◯	◯	**CHOOSE ONE** ◯ 1 slice or less **OR** ◯ 2 slices **OR** ◯ 3 slices or more
**Other Bread** (such as mixed grain, oat bran, raisin bread)	◯	◯	◯	◯	◯	◯	◯	◯	**CHOOSE ONE** ◯ 1 slice or less **OR** ◯ 2 slices **OR** ◯ 3 slices or more
**Rolls, Buns, Biscuits, or Flour Tortillas** (includes bagels, English muffins)	◯	◯	◯	◯	◯	◯	◯	◯	**CHOOSE ONE** ◯ 1 item or less **OR** ◯ 2 items or 1 bagel or English muffin **OR** ◯ 3 items or more
**Corn Tortillas, Corn Muffins, or Cornbread** (includes cornbread stuffing)	◯	◯	◯	◯	◯	◯	◯	◯	**CHOOSE ONE** ◯ 1 tortilla or 1 piece cornbread or 1/2 cup stuffing **OR** ◯ 2 tortillas or 1 muffin **OR** ◯ 3 tortillas or 2 muffins or more
**Bran, Blueberry or Other Muffins, Banana or Mango Bread**	◯	◯	◯	◯	◯	◯	◯	◯	**CHOOSE ONE** ◯ 1 regular muffin or 1 slice **OR** ◯ 1 large muffin or 2 slices **OR** ◯ 3 muffins or slices or more
**Sweet Rolls, Croissants, Doughnuts, Danish Pastry, or Coffee Cake**	◯	◯	◯	◯	◯	◯	◯	◯	**CHOOSE ONE** ◯ 1 item or less **OR** ◯ 2 items **OR** ◯ 3 items or more
**Pancakes, Waffles, or French Toast**	◯	◯	◯	◯	◯	◯	◯	◯	**CHOOSE ONE** ◯ 1 item or less **OR** ◯ 2 items **OR** ◯ 3 items or more
**Margarine Added to Bread Items**	◯	◯	◯	◯	◯	◯	◯	◯	**CHOOSE ONE** ◯ spread thin **OR** ◯ spread thick
**Butter Added to Bread Items**	◯	◯	◯	◯	◯	◯	◯	◯	**CHOOSE ONE** ◯ spread thin **OR** ◯ spread thick
**Peanut Butter Added to Bread Items**	◯	◯	◯	◯	◯	◯	◯	◯	**CHOOSE ONE** ◯ spread thin **OR** ◯ spread thick
**Jam or Jelly Added to Bread Items**	◯	◯	◯	◯	◯	◯	◯	◯	**CHOOSE ONE** ◯ spread thin **OR** ◯ spread thick
**Mayonnaise in Sandwiches**	◯	◯	◯	◯	◯	◯	◯	◯	**CHOOSE ONE** ◯ spread thin **OR** ◯ spread thick

BREAKFAST CEREALS, MILK, AND CHEESE	AVERAGE USE DURING LAST YEAR								YOUR USUAL SERVING SIZE
	Never or hardly ever	Once a month	2 to 3 times a month	Once a week	2 to 3 times a week	4 to 6 times a week	Once a day	2 or more times a day	
**Highly Fortified Cereals** (such as Product 19, Total, Most)	○	○	○	○	○	○	○	○	CHOOSE ONE<br>○ 1/2 cup or less **OR**<br>○ 1 cup or individual box **OR**<br>○ 1-1/2 cups or more
**Bran or High Fiber Cereals**	○	○	○	○	○	○	○	○	CHOOSE ONE<br>○ 1/2 cup or less **OR**<br>○ 1 cup or individual box **OR**<br>○ 1-1/2 cups or more
**Other Cold Cereals** (such as corn flakes, Cheerios, granola)	○	○	○	○	○	○	○	○	CHOOSE ONE<br>○ 1/2 cup or less **OR**<br>○ 1 cup or individual box **OR**<br>○ 1-1/2 cups or more
**Cooked Cereals** (such as oatmeal, cream of wheat, corn grits)	○	○	○	○	○	○	○	○	CHOOSE ONE<br>○ 1/2 cup or less **OR**<br>○ 1 cup or individual packet **OR**<br>○ 1-1/2 cups or more
**Whole Milk** (as beverage or added to cereal)	○	○	○	○	○	○	○	○	CHOOSE ONE<br>○ 1/2 cup or less **OR**<br>○ 1 cup or half-pint carton **OR**<br>○ 2 cups or more
**Lowfat Milk (1% or 2%)** (as beverage or added to cereal - includes lactaid and acidophilus milk)	○	○	○	○	○	○	○	○	CHOOSE ONE<br>○ 1/2 cup or less **OR**<br>○ 1 cup or half-pint carton **OR**<br>○ 2 cups or more
**Nonfat or Skim Milk or Buttermilk** (as beverage or added to cereal)	○	○	○	○	○	○	○	○	CHOOSE ONE<br>○ 1/2 cup or less **OR**<br>○ 1 cup or half-pint carton **OR**<br>○ 2 cups or more
**Yogurt** (includes lowfat and nonfat)	○	○	○	○	○	○	○	○	CHOOSE ONE<br>○ 1/2 cup or 4-6 oz. carton **OR**<br>○ 1 cup or 8 oz. carton **OR**<br>○ 2 cups or more
**Chocolate Milk, Cocoa, or Ovaltine**	○	○	○	○	○	○	○	○	CHOOSE ONE<br>○ 1/2 cup or less **OR**<br>○ 1 cup **OR**<br>○ 2 cups or more
**Milkshakes or Malts**	○	○	○	○	○	○	○	○	CHOOSE ONE<br>○ 1/2 milkshake or malt **OR**<br>○ 1 milkshake or malt (12 oz.) **OR**<br>○ 2 milkshakes or malts
**Cottage Cheese** (includes farmer's and ricotta cheese)	○	○	○	○	○	○	○	○	CHOOSE ONE<br>○ 1/4 cup or less **OR**<br>○ 1/2 cup or 1 scoop **OR**<br>○ 1 cup or more
**Lowfat Cheese** (such as lowfat American, lowfat Swiss, mozzarella)	○	○	○	○	○	○	○	○	CHOOSE ONE<br>○ 1/2 slice **OR**<br>○ 1 slice (1 ounce) **OR**<br>○ 2 slices (2 ounces) or more
**Other Cheese** (such as American, cheddar, cream cheese)	○	○	○	○	○	○	○	○	CHOOSE ONE<br>○ 1/2 slice or 1 Tablespoon **OR**<br>○ 1 slice (1 ounce) **OR**<br>○ 2 slices (2 ounces) or more

DESSERTS AND SNACKS	AVERAGE USE DURING LAST YEAR								YOUR USUAL SERVING SIZE
	Never or hardly ever	Once a month	2 to 3 times a month	Once a week	2 to 3 times a week	4 to 6 times a week	Once a day	2 or more times a day	
**Ice Cream**	◯	◯	◯	◯	◯	◯	◯	◯	**CHOOSE ONE**<br>◯ 1 scoop (1/2 cup) or less **OR**<br>◯ 2 scoops (1 cup) or 1 bar **OR**<br>◯ 3 to 4 scoops (1 pint) or more
**Ice Milk, Frozen Yogurt, or Sherbet**	◯	◯	◯	◯	◯	◯	◯	◯	**CHOOSE ONE**<br>◯ 1 scoop (1/2 cup) or less **OR**<br>◯ 2 scoops (1 cup) or 1 bar **OR**<br>◯ 3 to 4 scoops (1 pint) or more
**Cookies, Brownies, or Fruit Bars**	◯	◯	◯	◯	◯	◯	◯	◯	**CHOOSE ONE**<br>◯ 1 to 2 average size cookies **OR**<br>◯ 3 to 4 average or 1 extra large cookie or 1 brownie or fruit bar **OR**<br>◯ 2 large cookies or brownies or more
**Cake**	◯	◯	◯	◯	◯	◯	◯	◯	**CHOOSE ONE**<br>◯ 1 small piece or cupcake **OR**<br>◯ 1 average piece (1/12 of cake) **OR**<br>◯ 2 pieces or more
**Apple or Other Fruit Pies, Tarts, Cobblers, or Turnovers**	◯	◯	◯	◯	◯	◯	◯	◯	**CHOOSE ONE**<br>◯ 1 small piece **OR**<br>◯ 1 piece (1/8 pie) or 1 item **OR**<br>◯ 1/6 pie or more
**Pumpkin, Sweet Potato, or Carrot Pies**	◯	◯	◯	◯	◯	◯	◯	◯	**CHOOSE ONE**<br>◯ 1 small piece **OR**<br>◯ 1 average piece (1/8 pie) **OR**<br>◯ 1/6 pie or more
**Cream or Custard Pies, Eclairs, or Cream Puffs**	◯	◯	◯	◯	◯	◯	◯	◯	**CHOOSE ONE**<br>◯ 1 small piece **OR**<br>◯ 1 average piece or 1 item **OR**<br>◯ 1/6 pie or more
**Puddings or Custards** (includes flan)	◯	◯	◯	◯	◯	◯	◯	◯	**CHOOSE ONE**<br>◯ 1 snack-size or 1/2 cup **OR**<br>◯ 2 snack-size or 1 cup **OR**<br>◯ 3 snack-size or 1-1/2 cups
**Chocolate Candy**	◯	◯	◯	◯	◯	◯	◯	◯	**CHOOSE ONE**<br>◯ 1 to 3 pieces **OR**<br>◯ 1 regular-size bar **OR**<br>◯ 1 giant-size bar or more
**Dim Sum, such as Bao or Manapua** (Chinese bun with meat and vegetables)	◯	◯	◯	◯	◯	◯	◯	◯	**CHOOSE ONE**<br>◯ 1/2 bao or less **OR**<br>◯ 1 bao **OR**<br>◯ 2 bao or more
**Other Dim Sum** (such as pork hash, gau gee, fried won ton, eggroll)	◯	◯	◯	◯	◯	◯	◯	◯	**CHOOSE ONE**<br>◯ 1 to 2 pieces **OR**<br>◯ 3 to 4 pieces **OR**<br>◯ 5 pieces or more
**Crackers and Pretzels** (such as soda, graham, Japanese rice crackers, wheat thins)	◯	◯	◯	◯	◯	◯	◯	◯	**CHOOSE ONE**<br>◯ 4 to 5 snack or 1 large cracker **OR**<br>◯ 6 to 10 snack or 2 large crackers **OR**<br>◯ 3 large crackers or more
**Peanuts or Other Nuts**	◯	◯	◯	◯	◯	◯	◯	◯	**CHOOSE ONE**<br>◯ 12 nuts or less **OR**<br>◯ 1/4 cup **OR**<br>◯ 1/2 cup or more

For EACH FOOD GROUP, fill in the circle ◯ that best describes HOW OFTEN you ate those items during the last year. Then fill in the circle ◯ that best describes your USUAL SERVING SIZE.

SNACKS (continued)	AVERAGE USE DURING LAST YEAR: Never or hardly ever	Once a month	2 to 3 times a month	Once a week	2 to 3 times a week	4 to 6 times a week	Once a day	2 or more times a day	YOUR USUAL SERVING SIZE
**Potato, Corn, Tortilla or Other Chips, or Chicharrones** (pork rinds)	◯	◯	◯	◯	◯	◯	◯	◯	**CHOOSE ONE**<br>◯ 1 snack bag or 1/2 cup **OR**<br>◯ 1-ounce bag (1 cup) **OR**<br>◯ 1/2 twin-pack or more
**Popcorn**	◯	◯	◯	◯	◯	◯	◯	◯	**CHOOSE ONE**<br>◯ 1 to 3 cups or less **OR**<br>◯ 1 microwave bag **OR**<br>◯ 1 medium theater tub or more

ALCOHOLIC AND OTHER BEVERAGES	AVERAGE USE DURING LAST YEAR: Never or hardly ever	Once a month	2 to 3 times a month	Once a week	2 to 3 times a week	4 to 6 times a week	Once a day	2 to 3 times a day	4 or more times a day	YOUR USUAL SERVING SIZE
**Regular or Draft Beer**	◯	◯	◯	◯	◯	◯	◯	◯	◯	**CHOOSE ONE**<br>◯ 1 can or bottle or less **OR**<br>◯ 2 cans or bottles **OR**<br>◯ 3 cans or bottles **OR**<br>◯ 4 cans or bottles or more
**Light Beer**	◯	◯	◯	◯	◯	◯	◯	◯	◯	**CHOOSE ONE**<br>◯ 1 can or bottle or less **OR**<br>◯ 2 cans or bottles **OR**<br>◯ 3 cans or bottles **OR**<br>◯ 4 cans or bottles or more
**White or Pink Wine** (includes champagne and sake)	◯	◯	◯	◯	◯	◯	◯	◯	◯	**CHOOSE ONE**<br>◯ 1 glass or less **OR**<br>◯ 2 glasses **OR**<br>◯ 3 glasses **OR**<br>◯ 4 glasses or more
**Red Wine**	◯	◯	◯	◯	◯	◯	◯	◯	◯	**CHOOSE ONE**<br>◯ 1 glass or less **OR**<br>◯ 2 glasses **OR**<br>◯ 3 glasses **OR**<br>◯ 4 glasses or more
**Hard Liquor** (such as bourbon, scotch, gin, vodka, tequila, rum, cocktails)	◯	◯	◯	◯	◯	◯	◯	◯	◯	**CHOOSE ONE**<br>◯ 1 drink or less **OR**<br>◯ 2 drinks **OR**<br>◯ 3 drinks **OR**<br>◯ 4 drinks or more
**Regular Sodas** (such as Coca-Cola, Pepsi, 7-Up)	◯	◯	◯	◯	◯	◯	◯	◯	◯	**CHOOSE ONE**<br>◯ 1/2 can or small glass **OR**<br>◯ 1 can or large glass **OR**<br>◯ 2 cans or glasses **OR**<br>◯ 3 cans or glasses or more
**Diet Sodas** (such as Diet Coke, Diet Pepsi, Diet 7-Up)	◯	◯	◯	◯	◯	◯	◯	◯	◯	**CHOOSE ONE**<br>◯ 1/2 can or small glass **OR**<br>◯ 1 can or large glass **OR**<br>◯ 2 cans or glasses **OR**<br>◯ 3 cans or glasses or more

OTHER BEVERAGES	AVERAGE USE DURING LAST YEAR									WHAT DID YOU USUALLY ADD?
	Never or hardly ever	Once a month	2 to 3 times a month	Once a week	2 to 3 times a week	4 to 6 times a week	Once a day	2 to 3 times a day	4 or more times a day	
**Cappuccino - 1 cup or mug** (includes café au lait, caffé latte, café con leche)										**MARK ALL THAT APPLY**<br>Sugar or honey<br>Sugar substitute
**Regular Coffee - 1 cup or mug** (brewed or instant)										**MARK ALL THAT APPLY**<br>Cream or half & half<br>Milk<br>Non-dairy cream<br>Sugar or honey<br>Sugar substitute
**Decaffeinated ("Decaf") Coffee - 1 cup or mug** (brewed or instant)										**MARK ALL THAT APPLY**<br>Cream or half & half<br>Milk<br>Non-dairy cream<br>Sugar or honey<br>Sugar substitute
**Black Tea - 1 cup or glass** (such as Lipton's, oolong, iced tea)										**MARK ALL THAT APPLY**<br>Cream or half & half<br>Milk<br>Non-dairy cream<br>Sugar or honey<br>Sugar substitute
**Green, Herbal, or Other Tea - 1 cup**										
**Fortified Diet Beverages - 1 glass or can** (such as Slimfast)										

HOW OFTEN DID YOU EAT THE FOLLOWING ITEMS?	AVERAGE USE DURING LAST YEAR							
	Never or hardly ever	Once a month	2 to 3 times a month	Once a week	2 to 3 times a week	4 to 6 times a week	Once a day	2 or more times a day
**Western Pickles or Relish** (such as dill or sweet pickles)								
**Olives**								
**Salsa or Hot Chili Peppers** (red or green)								
**Garlic**								
**Onions**								
**Oriental Salted or Pickled Vegetables** (such as salted cabbage or leafy greens, takuwan, kim chee)								
**Seaweed** (fresh or dried) (such as ogo limu, furikake)								
**Gravy on meat, potatoes, rice**								

HOW OFTEN DID YOU ADD THE FOLLOWING ITEMS TO YOUR FOODS AT THE TABLE. . .	AVERAGE USE DURING LAST YEAR							
	Never or hardly ever	Once a month	2 to 3 times a month	Once a week	2 to 3 times a week	4 to 6 times a week	Once a day	2 or more times a day
Salt	○	○	○	○	○	○	○	○
Shoyu (Soy Sauce) or Teriyaki Sauce	○	○	○	○	○	○	○	○
Mustard	○	○	○	○	○	○	○	○
Catsup	○	○	○	○	○	○	○	○
Sour Cream	○	○	○	○	○	○	○	○

HOW OFTEN DID YOU EAT YOUR MEAT, POULTRY, OR FISH PREPARED IN THE FOLLOWING WAYS. . .	Never or hardly ever	Once a month	2 to 3 times a month	Once a week	2 to 3 times a week	4 to 6 times a week	Once a day	2 or more times a day
Charcoal-broiled	○	○	○	○	○	○	○	○
Oven-broiled	○	○	○	○	○	○	○	○
Fried	○	○	○	○	○	○	○	○
Barbecued	○	○	○	○	○	○	○	○

HOW OFTEN DID YOU EAT MEAT, CHICKEN, OR FISH COOKED WITH. . .	AVERAGE USE DURING LAST YEAR							
	Never or hardly ever	Once a month	2 to 3 times a month	Once a week	2 to 3 times a week	4 to 6 times a week	Once a day	2 or more times a day
Vegetable Oil	○	○	○	○	○	○	○	○
Salt Pork, Lard, or Bacon Fat	○	○	○	○	○	○	○	○
Vegetable Shortening (such as Crisco)	○	○	○	○	○	○	○	○
Margarine	○	○	○	○	○	○	○	○
Butter	○	○	○	○	○	○	○	○
Vegetable spray, water, or non-stick pan	○	○	○	○	○	○	○	○

ANSWER THE FOLLOWING FOR THE LAST YEAR:

WHEN YOU ATE MEAT, HOW WAS IT USUALLY PREPARED?
- ○ Rare
- ○ Medium
- ○ Well-done
- ○ Don't eat meat

WHEN YOU ATE MEAT, DID YOU EAT THE FAT:
- ○ Most of the time
- ○ Some of the time
- ○ Never or hardly ever
- ○ Don't eat meat

WHEN YOU ATE CHICKEN, DID YOU EAT THE SKIN:
- ○ Most of the time
- ○ Some of the time
- ○ Never or hardly ever
- ○ Don't eat chicken

WHAT KIND OF MARGARINE DID YOU USUALLY USE? (mark only one)
- ○ Regular Stick **OR**
- ○ Regular Tub **OR**
- ○ Diet or Spread **OR**
- ○ Don't use margarine
- ○ Don't know

WHAT KIND OF BUTTER DID YOU USUALLY USE? (mark only one)
- ○ Regular **OR**
- ○ Whipped **OR**
- ○ Don't use butter
- ○ Don't know

WHAT KIND OF VEGETABLE OIL DID YOU USUALLY USE? (mark only one)
- ○ Soybean or corn oil
- ○ Olive oil
- ○ Canola oil
- ○ Any other oil
- ○ Don't use oil
- ○ Don't know

VITAMINS AND MINERALS

25. DID YOU TAKE ANY MULTIVITAMINS OR MULTIVITAMINS WITH MINERALS DURING THE LAST YEAR *(at least once a week)*?

- ○ No
- ○ Yes → **IF YES, HOW MANY VITAMIN TABLETS DID YOU TAKE?**
  - ○ 1 to 3 a week
  - ○ 4 to 6 a week
  - ○ 1 a day
  - ○ 2 a day
  - ○ 3 or more a day

→ **IF YES, HOW MANY YEARS HAVE YOU TAKEN THEM?**
- ○ 1 year or less
- ○ 2 to 4 years
- ○ 5 years or more

26. DID YOU TAKE ANY OF THE FOLLOWING VITAMINS OR MINERALS BY ITSELF DURING THE LAST YEAR *(at least once a week)*?

		IF YES, HOW MANY TABLETS DID YOU TAKE?	IF YES, HOW MANY YEARS HAVE YOU TAKEN THEM?	IF YES, WHAT WAS THE DOSE PER TABLET?
**VITAMIN A *(BY ITSELF)***	○ No<br>○ Yes	○ 1 to 3 a week<br>○ 4 to 6 a week<br>○ 1 a day<br>○ 2 a day<br>○ 3 or more a day	○ 1 year or less<br>○ 2 to 4 years<br>○ 5 years or more	○ 5,000 I.U. (International Units) or less<br>○ 6,000 to 10,000 I.U.<br>○ 11,000 to 24,000 I.U.<br>○ 25,000 I.U. or more<br>○ Don't know
**VITAMIN C *(BY ITSELF)***	○ No<br>○ Yes	○ 1 to 3 a week<br>○ 4 to 6 a week<br>○ 1 a day<br>○ 2 a day<br>○ 3 or more a day	○ 1 year or less<br>○ 2 to 4 years<br>○ 5 years or more	○ 250 mg (milligrams) or less<br>○ 300 to 500 mg.<br>○ 600 to 4,000 mg.<br>○ 5,000 to 9,000 mg.<br>○ 10,000 mg. or more<br>○ Don't know
**VITAMIN E *(BY ITSELF)***	○ No<br>○ Yes	○ 1 to 3 a week<br>○ 4 to 6 a week<br>○ 1 a day<br>○ 2 a day<br>○ 3 or more a day	○ 1 year or less<br>○ 2 to 4 years<br>○ 5 years or more	○ 200 I.U. (International Units) or less<br>○ 250 to 800 I.U.<br>○ 825 to 1,200 I.U.<br>○ 1,250 I.U. or more<br>○ Don't know
**BETA-CAROTENE *(BY ITSELF)***	○ No<br>○ Yes	○ 1 to 3 a week<br>○ 4 to 6 a week<br>○ 1 a day<br>○ 2 a day<br>○ 3 or more a day	○ 1 year or less<br>○ 2 to 4 years<br>○ 5 years or more	○ 6,000 mcg (micrograms) or less<br>○ 7,000 to 15,000 mcg.<br>○ 16,000 mcg. or more<br>○ Don't know
**CALCIUM *(BY ITSELF)***	○ No<br>○ Yes	○ 1 to 3 a week<br>○ 4 to 6 a week<br>○ 1 a day<br>○ 2 a day<br>○ 3 or more a day	○ 1 year or less<br>○ 2 to 4 years<br>○ 5 years or more	○ 250 mg (milligrams) or less<br>○ 300 to 600 mg.<br>○ 625 to 1,000 mg.<br>○ 1,250 mg. or more<br>○ Don't know
**SELENIUM *(BY ITSELF)***	○ No<br>○ Yes	○ 1 to 3 a week<br>○ 4 to 6 a week<br>○ 1 a day<br>○ 2 a day<br>○ 3 or more a day	○ 1 year or less<br>○ 2 to 4 years<br>○ 5 years or more	○ 75 mcg. (micrograms) or less<br>○ 100 to 150 mcg.<br>○ 200 to 225 mcg.<br>○ 250 mcg. or more<br>○ Don't know
**IRON *(BY ITSELF)***	○ No<br>○ Yes	○ 1 to 3 a week<br>○ 4 to 6 a week<br>○ 1 a day<br>○ 2 a day<br>○ 3 or more a day	○ 1 year or less<br>○ 2 to 4 years<br>○ 5 years or more	○ 50 mg. (milligrams) or less<br>○ 51 to 150 mg.<br>○ 151 mg. or more<br>○ Don't know

MEDICATION HISTORY

27. HAVE YOU EVER TAKEN ANY OF THE FOLLOWING MEDICATIONS AT LEAST TWO TIMES PER WEEK *(for one month or longer)*?

Medication		IF YES, HOW MANY YEARS HAVE YOU EVER TAKEN THEM?
**Aspirin** (Anacin, Bufferin, Bayer, Excedrin, or other)	○ No<br>○ Yes, but not at this time →<br>○ Yes, currently →	○ 1 year or less ○ 6 to 10 years<br>○ 2 to 3 years ○ 11 years or more<br>○ 4 to 5 years
**Acetaminophen** (Tylenol, Anacin-3, Panadol, or other)	○ No<br>○ Yes, but not at this time →<br>○ Yes, currently	○ 1 year or less ○ 6 to 10 years<br>○ 2 to 3 years ○ 11 years or more<br>○ 4 to 5 years
**Other Pain Relief Medication** (Motrin, Nuprin, Advil, Indocin, Naprosyn, Medipren, Ibuprofen, or other)	○ No<br>○ Yes, but not at this time →<br>○ Yes, currently	○ 1 year or less ○ 6 to 10 years<br>○ 2 to 3 years ○ 11 years or more<br>○ 4 to 5 years
**Water Pills for High Blood Pressure or other Reasons** (Diuril, Hydrodiuril, Dyazide, or other)	○ No<br>○ Yes, but not at this time →<br>○ Yes, currently	○ 1 year or less ○ 6 to 10 years<br>○ 2 to 3 years ○ 11 years or more<br>○ 4 to 5 years
**Reserpine** (Raudixin, Ser-Ap-Es, Hydropres, Rauwolfia, Metatensin, or other)	○ No<br>○ Yes, but not at this time →<br>○ Yes, currently	○ 1 year or less ○ 6 to 10 years<br>○ 2 to 3 years ○ 11 years or more<br>○ 4 to 5 years
**Other Blood Pressure Medication** (Aldomet, Hygroton, Minipress, Cardizem, Procardia, Vasotec, or other)	○ No<br>○ Yes, but not at this time →<br>○ Yes, currently	○ 1 year or less ○ 6 to 10 years<br>○ 2 to 3 years ○ 11 years or more<br>○ 4 to 5 years
**Antacids** (Tums, Rolaids, Digel, Maalox, Gelusil, Alka-Seltzer, or other)	○ No<br>○ Yes, but not at this time →<br>○ Yes, currently	○ 1 year or less ○ 6 to 10 years<br>○ 2 to 3 years ○ 11 years or more<br>○ 4 to 5 years
**Tagamet, Zantac, or Pepcid for Peptic Ulcer**	○ No<br>○ Yes, but not at this time →<br>○ Yes, currently	○ 1 year or less ○ 6 to 10 years<br>○ 2 to 3 years ○ 11 years or more<br>○ 4 to 5 years
**Allergy Pills or Shots** (Antihistamines)	○ No<br>○ Yes, but not at this time →<br>○ Yes, currently	○ 1 year or less ○ 6 to 10 years<br>○ 2 to 3 years ○ 11 years or more<br>○ 4 to 5 years
**Laxatives**	○ No<br>○ Yes, but not at this time →<br>○ Yes, currently	○ 1 year or less ○ 6 to 10 years<br>○ 2 to 3 years ○ 11 years or more<br>○ 4 to 5 years

WORK HISTORY

28. IN WHICH OCCUPATIONAL CATEGORY HAVE YOU WORKED THE LONGEST? *(Mark only one)*

○ Laborer or Farm Worker
○ Factory Worker or Machine Operator
○ Clerical or Office Worker
○ Sales
○ Manager or Administrator
○ Craftsperson
○ Small Business Owner
○ Professional/Technical
○ Unemployed
○ Other *(write in)*

29. PLEASE MARK ANY OF THE FOLLOWING INDUSTRIES OR OCCUPATIONS IN WHICH YOU WERE EMPLOYED FOR 10 YEARS OR LONGER. *(You may mark more than one.)*

○ Metal production or processing
○ Mining, quarrying, rock crushing, or cement manufacturing
○ Cotton, wool, or textile processing
○ Plastic production or processing
○ Gasoline refining or distribution
○ Chemical production or use
○ Rubber or tire manufacturing
○ Shipyard work
○ Farming
○ Furniture making or woodworking
○ Automotive repair
○ Pesticide production
○ Paint production or use
○ None of these

PHYSICAL ACTIVITY

30. ON THE AVERAGE, DURING THE LAST YEAR, HOW MANY HOURS *IN A DAY* DID YOU SLEEP (include naps)?

- ○ 5 hours or less
- ○ 6 hours
- ○ 7 hours
- ○ 8 hours
- ○ 9 hours
- ○ 10 hours or more

31. ON THE AVERAGE, DURING THE LAST YEAR, HOW MANY HOURS *IN A DAY* DID YOU SPEND IN THE FOLLOWING SITTING ACTIVITIES?

	NEVER	Less than 1 hr.	1 to 2 hrs.	3 to 4 hrs.	5 to 6 hrs.	7 to 10 hrs.	11 hrs. or more
**Sitting in car or bus**	○	○	○	○	○	○	○
**Sitting at work**	○	○	○	○	○	○	○
**Watching TV**	○	○	○	○	○	○	○
**Sitting at meals**	○	○	○	○	○	○	○
**Other sitting activities** (such as reading, playing cards, sewing)	○	○	○	○	○	○	○

32. ON THE AVERAGE, DURING THE LAST YEAR, HOW MANY HOURS *IN A WEEK* DID YOU SPEND IN THE FOLLOWING ACTIVITIES?

	NEVER	1/2 to 1 hr.	2 to 3 hrs.	4 to 6 hrs.	7 to 10 hrs.	11 to 20 hrs.	21 to 30 hrs.	31 hrs. or more
**Strenuous Sports** (such as jogging, bicycling on hills, tennis, racquetball, swimming laps, aerobics)	○	○	○	○	○	○	○	○
**Vigorous Work** (such as moving heavy furniture, loading or unloading trucks, shoveling, weight lifting, or equivalent manual labor)	○	○	○	○	○	○	○	○
**Moderate Activity** (such as housework, brisk walking, golfing, bowling, bicycling on level ground, gardening)	○	○	○	○	○	○	○	○

33. ON THE AVERAGE, DURING THE LAST YEAR, HOW MANY *TIMES A WEEK* DID YOU TAKE PART IN VIGOROUS PHYSICAL ACTIVITY (STRENUOUS SPORTS OR WORK) LONG ENOUGH TO WORK UP A SWEAT?

- ○ NEVER
- ○ 1 time
- ○ 2 times
- ○ 3 times
- ○ 4 times
- ○ 5 times
- ○ 6 times
- ○ 7 times or more

MEN, PLEASE GO TO PAGE 26

WOMEN, PLEASE GO TO NEXT PAGE

34. HOW OLD WERE YOU WHEN YOU HAD YOUR FIRST MENSTRUAL PERIOD?

- ◯ Less than 11 years
- ◯ 11 - 12 years
- ◯ 13 - 14 years
- ◯ 15 - 16 years
- ◯ 17 years or older

35. HOW OLD WERE YOU WHEN YOUR PERIODS BECAME REGULAR (ABOUT ONCE A MONTH)?

- ◯ Less than 11 years
- ◯ 11 - 12 years
- ◯ 13 - 14 years
- ◯ 15 - 16 years
- ◯ 17 years or older
- ◯ Never became regular

36. HAVE YOU EVER BEEN PREGNANT?

- ◯ No *(go to question 37)*
- ◯ Yes

IF YES, WHAT WAS THE OUTCOME OF YOUR *FIRST* PREGNANCY?

- ◯ Live birth
- ◯ Stillbirth
- ◯ Tubal pregnancy
- ◯ Miscarriage
- ◯ Induced abortion

IF YOU EVER HAD CHILDREN, HOW OLD WERE YOU WHEN YOUR *FIRST* CHILD WAS BORN?

- ◯ Less than 15 years
- ◯ 15 - 17 years
- ◯ 18 - 20 years
- ◯ 21 - 25 years
- ◯ 26 - 30 years
- ◯ 31 - 35 years
- ◯ 36 years or older

37. WHAT ONE METHOD OF CONTRACEPTION HAVE YOU USED FOR THE LONGEST TIME?

- ◯ Birth control pills
- ◯ Birth control injections
- ◯ Condom
- ◯ Diaphragm
- ◯ Spermicide
- ◯ IUD
- ◯ Partner has been sterilized (vasectomy)
- ◯ I have been sterilized (tubes tied)
- ◯ Withdrawal
- ◯ None of the above

38. DID YOU EVER TAKE BIRTH CONTROL PILLS FOR ONE MONTH OR LONGER?

- ◯ No *(go to question 39)*
- ◯ Yes, and I am currently taking them
- ◯ Yes, but I no longer take them

IF YES, HOW OLD WERE YOU WHEN YOU STARTED TAKING THEM?

- ◯ Less than 15 years
- ◯ 15 - 17 years
- ◯ 18 - 20 years
- ◯ 21 - 25 years
- ◯ 26 - 30 years
- ◯ 31 - 35 years
- ◯ 36 years or older

IF YES, HOW MANY YEARS DID YOU TAKE THEM IN TOTAL?

- ◯ Less than one year
- ◯ 1 - 2 years
- ◯ 3 - 5 years
- ◯ 6 - 9 years
- ◯ 10 - 14 years
- ◯ 15 - 19 years
- ◯ 20 years or more

39. HAVE YOU EVER HAD A HYSTERECTOMY (complete removal of the uterus)?

- ◯ No
- ◯ Yes

40. HAVE YOU EVER HAD YOUR OVARIES REMOVED?

- ◯ No
- ◯ Yes, one only
- ◯ Yes, both ovaries
- ◯ Yes, don't know how many
- ◯ Don't know

41. HAVE YOUR MENSTRUAL PERIODS STOPPED PERMANENTLY?

- ◯ No *(go to question 42)*
- ◯ Yes

IF YES, HOW OLD WERE YOU WHEN THIS HAPPENED?

- ◯ Less than 40 years
- ◯ 40 - 44 years
- ◯ 45 - 49 years
- ◯ 50 - 54 years
- ◯ 55 years or older

IF YES, FOR WHAT REASON?

- ◯ Natural menopause
- ◯ Surgery
- ◯ Radiation
- ◯ Medication

42. DID YOU EVER TAKE ESTROGEN (FEMALE HORMONES) BY PILL, INJECTION, OR PATCH FOR MENOPAUSE OR OTHER REASONS?

- ◯ No *(go to question 44)*
- ◯ Yes, and I am currently taking it
- ◯ Yes, but I no longer take it

IF YES, HOW OLD WERE YOU WHEN YOU STARTED TAKING ESTROGEN?

- ◯ Less than 40 years
- ◯ 40 - 44 years
- ◯ 45 - 49 years
- ◯ 50 - 54 years
- ◯ 55 - 59 years
- ◯ 60 years or older

IF YES, HOW MANY YEARS DID YOU TAKE ESTROGEN?

- ◯ Less than one year
- ◯ 1 - 2 years
- ◯ 3 - 5 years
- ◯ 6 - 9 years
- ◯ 10 - 14 years
- ◯ 15 - 19 years
- ◯ 20 years or more

IF YOU USED AN ESTROGEN PILL, DID YOU EVER TAKE PREMARIN?

- ◯ No *(go to question 43)*
- ◯ Yes

IF YES, WHICH ONE OF THE FIVE PREMARIN PILLS DID YOU TAKE *MOST OFTEN* ?

- ◯ Green (0.3 mg)
- ◯ Brown or red (0.625 mg)
- ◯ White (0.9 mg)
- ◯ Yellow or orange (1.25 mg)
- ◯ Purple (2.5 mg)

43. DID YOU EVER TAKE PROGESTERONE (SUCH AS PROVERA) ALONG WITH ESTROGEN FOR MENOPAUSE OR OTHER REASONS?

- ◯ No *(go to question 44)*
- ◯ Yes, and I am currently taking it
- ◯ Yes, but I no longer take it

IF YES, HOW OLD WERE YOU WHEN YOU STARTED TAKING PROGESTERONE?

- ◯ Less than 40 years
- ◯ 40 - 44 years
- ◯ 45 - 49 years
- ◯ 50 - 54 years
- ◯ 55 - 59 years
- ◯ 60 years or older

IF YES, HOW MANY YEARS DID YOU TAKE PROGESTERONE IN TOTAL?

- ◯ Less than one year
- ◯ 1 - 2 years
- ◯ 3 - 5 years
- ◯ 6 - 9 years
- ◯ 10 - 14 years
- ◯ 15 - 19 years
- ◯ 20 years or more

44. HAVE YOU EVER HAD ANY OF THE FOLLOWING TESTS? IF YES, PLEASE MARK HOW LONG IT HAS BEEN SINCE YOU LAST HAD THE TEST.

	Ever had?			Number of Years Since Last Test		
	No	Yes		Less than 1 year	1 to 2 yrs.	3 years or more
**Mammogram** (breast x-ray)	◯	◯	→	◯	◯	◯
**PAP smear**	◯	◯	→	◯	◯	◯

PLEASE GO TO NEXT PAGE

45. HAVE YOUR MOTHER OR FULL SISTERS EVER HAD ANY OF THE FOLLOWING CANCERS? ***(Include any who may have died.)***

		IF YES, WHO? →	IF YES, DID ANY OCCUR BEFORE AGE 50?
**BREAST CANCER**	○ No (or Don't Know) ○ Yes →	○ Mother ○ 1 sister ○ 2 or more sisters →	○ No ○ Yes ○ Don't know
**OVARIAN CANCER**	○ No (or Don't Know) ○ Yes →	○ Mother ○ 1 sister ○ 2 or more sisters →	○ No ○ Yes ○ Don't know
**COLON CANCER**	○ No (or Don't Know) ○ Yes →	○ Mother ○ 1 sister ○ 2 or more sisters →	○ No ○ Yes ○ Don't Know
**OTHER CANCER**	○ No (or Don't Know) ○ Yes → *(write in kind)*	○ Mother ○ 1 sister ○ 2 or more sisters →	○ No ○ Yes ○ Don't know

46. HAVE YOUR FATHER OR FULL BROTHERS EVER HAD ANY OF THE FOLLOWING CANCERS? ***(Include any who may have died.)***

		IF YES, WHO? →	IF YES, DID ANY OCCUR BEFORE AGE 50?
**PROSTATE CANCER**	○ No (or Don't Know) ○ Yes →	○ Father ○ 1 brother ○ 2 or more brothers →	○ No ○ Yes ○ Don't know
**COLON CANCER**	○ No (or Don't Know) ○ Yes →	○ Father ○ 1 brother ○ 2 or more brothers →	○ No ○ Yes ○ Don't know
**OTHER CANCER**	○ No (or Don't Know) ○ Yes → *(write in kind)*	○ Father ○ 1 brother ○ 2 or more brothers →	○ No ○ Yes ○ Don't know

47. WRITE IN YOUR BIRTHDATE AND FILL IN THE CIRCLES. ***(See example in instructions)***

MONTH	DAY		YEAR			
○ JAN			1	9		
○ FEB						
○ MAR	0	0			0	0
○ APR	1	1			1	1
○ MAY	2	2			2	2
○ JUN	3	3			3	3
○ JUL		4			4	4
○ AUG		5			5	5
○ SEP		6			6	6
○ OCT		7			7	7
○ NOV		8			8	8
○ DEC		9			9	9

48. PLEASE WRITE IN YOUR SOCIAL SECURITY NUMBER AND FILL IN THE CIRCLES

			–			–				
0	0	0		0	0		0	0	0	0
1	1	1		1	1		1	1	1	1
2	2	2		2	2		2	2	2	2
3	3	3		3	3		3	3	3	3
4	4	4		4	4		4	4	4	4
5	5	5		5	5		5	5	5	5
6	6	6		6	6		6	6	6	6
7	7	7		7	7		7	7	7	7
8	8	8		8	8		8	8	8	8
9	9	9		9	9		9	9	9	9

(This will only be used to identify you in this study)

49. PLEASE WRITE IN YOUR PHONE NUMBER AND FILL IN THE CIRCLES

Area Code				Number							
							–				
0	0	0		0	0	0		0	0	0	0
1	1	1		1	1	1		1	1	1	1
2	2	2		2	2	2		2	2	2	2
3	3	3		3	3	3		3	3	3	3
4	4	4		4	4	4		4	4	4	4
5	5	5		5	5	5		5	5	5	5
6	6	6		6	6	6		6	6	6	6
7	7	7		7	7	7		7	7	7	7
8	8	8		8	8	8		8	8	8	8
9	9	9		9	9	9		9	9	9	9

(We may need to get in touch with you to clarify certain answers on your survey)

IN CASE WE NEED TO CONTACT YOU IN THE FUTURE AND YOU HAVE MOVED, PLEASE GIVE THE NAME OF SOMEONE AT A DIFFERENT ADDRESS TO WHOM WE MIGHT WRITE FOR YOUR NEW LOCATION.

Name of relative or friend: ____________________ Relationship

His or her address: ____________________
Street City State Zip Code

His or her phone number (   ) ____________________
Area Code

Please check to see that ALL PAGES are completed. Place the questionnaire in the postage paid envelope provided and mail it to us.

THANK YOU AGAIN FOR YOUR TIME AND COOPERATION!
