# Supplementary material for: Sex and ethnic/racial-specific risk factors for gallbladder disease
Source: BMC Gastroenterol. 2017 Dec 8;17:153. doi: 10.1186/s12876-017-0678-6 (PMC5723039; doi:10.1186/s12876-017-0678-6)
Supplement: Supplementary file 2 — Distribution of Risk Factors among MEC participants by sex and race/ethnicity. This table shows the distribution of risk factors in the MEC by sex and race/ethnicity. (DOCX 16 kb) [file 12876_2017_678_MOESM2_ESM.docx]

**Additional file 2: Table S1. Distribution of Risk Factors among MEC participants by sex and race/ethnicity**

|  | **Men** | | | | | | **Women** | | | | | |
| --- | --- | --- | --- | --- | --- | --- | --- | --- | --- | --- | --- | --- |
|  | **White** | **African American** | **Native Hawaiian** | **Japanese American** | **Latino –US born** | **Latino –**  **Mex/SA** | **White** | **African American** | **Native Hawaiian** | **Japanese American** | **Latino –**  **US born** | **Latino – Mex/SA** |
| **No. of at risk** | 14,271 | 11,154 | 3,098 | 16,983 | 10,021 | 9,374 | 17,832 | 19,055 | 3,844 | 19,004 | 10,384 | 9,389 |
| **Age at cohort entry**  mean (SD) years | 59.9 (8.5) | 61.9 (8.8) | 57.6 (7.6) | 61.2 (8.6) | 61.3 (7.6) | 59.0 (7.8) | 59.7 (8.5) | 60.5 (9.1) | 57.5 (7.6) | 60.7 (8.4) | 60.3 (7.8) | 58.0 (7.6) |
| **Education**  High school or less  Some college  College graduate or more | 22.7%  29.4%  47.9% | 41.5%  36.4%  22.2% | 50.2%  31.2%  18.6% | 33.2%  31.3%  35.5% | 55.2%  29.8%  15.0% | 74.1%  17.1%  8.8% | 31.6%  33.2%  35.2% | 39.7%  37.6%  22.6% | 56.3%  28.4%  15.3% | 38.0%  30.7%  31.3% | 66.1%  24.3%  9.5% | 78.7%  14.4%  6.9% |
| **BMI at baseline (kg/m^2^)**  < 25  25 - < 30  ≥ 30 | 36.7%  46.5%  16.9% | 29.9%  47.7%  22.4% | 20.3%  44.6%  35.1% | 50.0%  42.6%  7.4% | 25.0%  52.2%  22.8% | 25.2%  55.2%  19.6% | 52.2%  29.2%  18.6% | 25.5%  36.9%  37.6% | 31.2%  33.7%  35.0% | 70.5%  23.5%  6.0% | 31.4%  37.2%  31.4% | 31.7%  43.0%  25.3% |
| **Diabetes**  No  Yes | 93.5%  6.5% | 83.8%  16.2% | 84.9%  15.1% | 88.2%  11.8% | 81.7%  18.3% | 85.5%  14.5% | 94.6%  5.4% | 84.8%  15.2% | 86.7%  13.3% | 91.5%  8.5% | 84.1%  15.9% | 86.6%  13.4% |
| **Smoking Status**  Never  Past  Current | 32.1%  52.2%  15.7% | 24.0%  49.4%  26.6% | 32.0%  47.3%  20.7% | 29.5%  56.3%  14.2% | 29.5%  53.2%  17.3% | 33.8%  47.8%  18.3% | 45.3%  38.5%  16.2% | 45.2%  34.8%  20.0% | 44.5%  33.5%  22.0% | 68.5%  22.7%  8.8% | 59.0%  29.0%  12.0% | 71.9%  19.7%  8.5% |
| **Alcohol Intake**  None  < 24 g/day  ≥ 24 g/day | 28.5%  45.5%  26.0% | 44.4%  39.9%  15.7% | 41.1%  39.5%  19.4% | 44.6%  40.4%  15.0% | 35.3%  46.4%  18.3% | 36.3%  50.1%  13.6% | 41.0%  47.5%  11.5% | 62.5%  32.8%  4.7% | 63.7%  31.9%  4.5% | 76.6%  21.8%  1.6% | 59.7%  37.1%  3.2% | 69.3%  29.4%  1.4% |
| **Vigorous Activity (hrs/day)**  0  > 0- ≤ 0.21  > 0.21- ≤ 0.46  > 0.46 | 28.4%  14.9%  16.5%  37.7% | 38.1%  15.8%  15.0%  26.6% | 19.1%  12.5%  18.1%  47.7% | 34.4%  18.7%  16.7%  27.8% | 30.9%  15.2%  16.3%  34.3% | 29.8%  13.9%  14.1%  36.2% | 49.7%  15.8%  12.7%  17.7% | 58.2%  16.7%  8.9%  8.8% | 41.7%  17.3%  15.3%  22.1% | 61.0%  15.7%  10.7%  9.4% | 56.6%  16.3%  10.6%  11.0% | 53.2%  14.5%  10.0%  11.6% |
